# Effective Search of Triterpenes with Anti-HSV-1 Activity Using a Classification Model by Logistic Regression

**DOI:** 10.3389/fchem.2021.763794

**Published:** 2021-11-02

**Authors:** Keiko Ogawa, Seikou Nakamura, Haruka Oguri, Kaori Ryu, Taichi Yoneda, Rumiko Hosoki

**Affiliations:** ^1^ Laboratory of Regulatory Science, College of Pharmaceutical Sciences, Ritsumeikan University, Kusatsu, Japan; ^2^ Department of Pharmacognosy, Kyoto Pharmaceutical University, Kyoto, Japan

**Keywords:** natural products, triterpene, saponin, herpes simplex virus type 1 (HSV-1), machine learing, QSAR

## Abstract

Natural products are an excellent source of skeletons for medicinal seeds. Triterpenes and saponins are representative natural products that exhibit anti-herpes simplex virus type 1 (HSV-1) activity. However, there has been a lack of comprehensive information on the anti-HSV-1 activity of triterpenes. Therefore, expanding information on the anti-HSV-1 activity of triterpenes and improving the efficiency of their exploration are urgently required. To improve the efficiency of the development of anti-HSV-1 active compounds, we constructed a predictive model for the anti-HSV-1 activity of triterpenes by using the information obtained from previous studies using machine learning methods. In this study, we constructed a binary classification model (i.e., active or inactive) using a logistic regression algorithm. As a result of the evaluation of predictive model, the accuracy for the test data is 0.79, and the area under the curve (AUC) is 0.86. Additionally, to enrich the information on the anti-HSV-1 activity of triterpenes, a plaque reduction assay was performed on 20 triterpenes. As a result, chikusetsusaponin IVa (**11**: IC_50_ = 13.06 *μ*M) was found to have potent anti-HSV-1 with three potentially anti-HSV-1 active triterpenes. The assay result was further used for external validation of predictive model. The prediction of the test compounds in the activity test showed a high accuracy (0.83) and AUC (0.81). We also found that this predictive model was found to be able to successfully narrow down the active compounds. This study provides more information on the anti-HSV-1 activity of triterpenes. Moreover, the predictive model can improve the efficiency of the development of active triterpenes by integrating many previous studies to clarify potential relationships.

## Introduction

HSV-1 is a common human pathogen (Arduino and Porter, 2008). According to a report from the World Health Organization, HSV-1 has been widespread and estimated to have infected 3.7 billion people globally (World Health Organization, 2020). The symptoms are usually benign; however, in some cases, severe conditions may occur with the development of herpetic encephalitis (Bradshaw and Venkatesan, 2016). Standard therapeutic drugs such as acyclovir, penciclovir, and vidarabine are all based on the nucleobase structure. Long-term prophylaxis and treatment with acyclovir or other nucleobase drugs has been reported to result in the development of resistance (Piret and Boivin, 2011). Therefore, searching for new anti-HSV-1 compounds with other structural characteristics is essential.

Our study group has been conducting research to discover new compounds (Kondo et al., 2020; Nakamura et al., 2021) and anti-HSV-1 active natural compounds (Ogawa et al., 2018; Yoneda et al., 2018). Triterpenes are known to contain a number of anti-HSV-1 compounds. In a previous study, Ikeda et al. evaluated the anti-HSV-1 activity of 15 oleanane triterpenes and reported several triterpenes such as glycyrrhizic acid methyl ester with an IC_50_ in the single-digit molar range (Ikeda et al., 2005). Baltina et al. evaluated the anti-HSV-1 activity of lupane triterpenes and proposed that simple structural modifications for lupane triterpenes should exhibit increased activity (Baltina et al., 2003). Hassan et al. reported that cucurbitacin B had strong anti-HSV-1 activity with high selectivity index (Hassan et al., 2017). The activity of triterpenes has been reported in many studies, especially related to the isolation reports of natural compounds (Lv et al., 2016; Isaka et al., 2017).

However, the structure-activity relationship (SAR) of anti-HSV-1 activity of triterpenes seems to be quite difficult. Wachsman pointed out that the SAR did not follow any clear pattern of brassinosteroids (Wachsman et al., 2004). In another study, a slight structural change can greatly affect anti-HSV-1 activity (Kinjo et al., 2000). Another limitation involves the inconsistent experimental protocol and the bias of test compounds, as most activity reports have been related to isolation reports. Although many studies have been conducted about triterpenes with HSV-1 assay, there are few reports that comprehensively examine the relationship between triterpene skeletons and their anti-HSV-1 activity. These backgrounds has presented difficulties in elucidating the relationship between triterpenes and anti-HSV-1 activity. Therefore, we attempted to develop the strategy to facilitate the narrowing down of anti-HSV-1 active compounds by integrating previously reported data using machine learning methods.

In recent years, quantitative structure–activity relationship (QSAR) studies have been successful in investigating the relationship between the structure and activity of compounds (Saiz-Urra et al., 2007; Masand et al., 2020). In QSAR studies, the structural features of compounds are converted into numerical values as molecular descriptors to analyze their relationship to activity. For instance, Saiz-Urra et al. described the antitumor activity of naphthoquinone ester derivatives using regression with 2D-autocorrelation descriptors.

In this study, to construct a predictive anti HSV-1 activity model, the problem to be solved is the difference in experimental conditions depending on the references. The results of the anti-HSV-1 activity assay were affected by experimental conditions, such as incubation time or cell type. Regression-based methods are commonly used and successfully perform activity prediction (Nagai et al., 2019; Kaushik et al., 2021). However, because these methods provide concrete activity values as output, they may contain large errors when using data with inconsistent experimental protocols.

Therefore, we decided to apply a binary classification model to predict the anti-HSV-1 activity. The classification model sets a threshold value and classifies the samples into two groups, “active” or “inactive”: “active” if the activity is stronger than the threshold value, and “inactive” if the activity is weaker than the threshold value. This method allowed us to demonstrate the basic trend of the anti-HSV-1 activity. Because it is only based on whether the activity is stronger or weaker than the threshold value, it minimizes the influence of differences in experimental conditions on the activity value.

In addition, we performed anti-HSV-1 assay to use for external-validation of constructed binary classification model and to expanding information on the anti-HSV-1 activity of triterpenes. Therefore, we selected 20 triterpenes randomly from a natural product library constructed by our study group and measured their anti-HSV-1 activity. Twenty triterpenes were initially isolated from three plants: *Pfaffia glomerata* (Amaranthaceae), *Inonotus obliquus* (Hymenochaetaceae), and *Isodon japonicus* (Lamiaceae). *P. glomerata* and *I. japonicus* have been reported to contain various types of triterpenes (Shiobara et al., 1993; Mroczek, 2015). However, they have not been studied for their anti-HSV-1 activity. *I. obliquus* has been reported to contain various triterpenes (Nakata et al., 2007; Ying et al., 2020) and its extracted form exhibited efficacy against HSV-1 (Polkovnikova et al., 2014). However, the active compounds have not yet been determined. We performed a plaque reduction assay on Vero cells to evaluate anti-HSV-1 activity of triterpenes. The assay results were further used for external validation of the constructed predictive model.

In this study, a predictive model for the anti-HSV-1 activity of triterpenes by logistic regression was constructed using previously reported data from databases and original studies. In addition, 20 triterpenes were examined for their anti-HSV-1 activity by using a plaque reduction assay. The predictive model was evaluated through cross-validation and validated by comparison with the activity test results. This study describes an approach to improve the efficiency of searching for active compounds by using machine learning integrating information from previous reports and databases, as well as a new report on the anti-HSV-1 activity of 20 triterpenes.

## Materials and Methods

### Data Collection

Triterpenes and their anti-HSV-1 activity were collected from two databases, namely, ChEMBL (Gaulton, 2017) and Dictionary of Natural Products (DNP) (Taylor and Francis Group, 2021), and 53 references were searched for on SciFinder. In the ChEMBL database, “plaque reduction assay,” “cytopathic effect,” and “CPE” were used as search words, and the activity data were downloaded as CSV files. The assay data related to HSV-1 were extracted by “Assay description” and “Target name” with “Herpes simplex virus (type 1/strain F)” (CHEMBL613200). A total of 76 triterpenes and their activities were obtained from the ChEMBL database. From the DNP, 14 triterpenes were collected using the search words “herpes” for biological use. We used SciFinder to look for references on anti-HSV-1 active triterpenes by using combinations of following words: “triterpene,” “saponin,” “steroid,” “herpes,” “HSV-1,” “isolated,” and “synthesis.” We carefully scrutinized the references and selected reliable results. The following information was retrieved from the reliable references: compound name, activity (IC_50_, EC_50_ or inhibition (%) with test concentration), virus strain, cell line, assay protocol, journal name, DOI, year of publication. A total of 472 triterpenes and their anti HSV-1 activity were obtained.

### Data Cleaning and Threshold Setting

All collected triterpenes were converted to the canonical SMILES format to identify duplicates. 76 triterpenes from the ChEMBL database contained SMILES information. The SMILES for the remaining triterpenes was created by obtaining the CAS numbers or mol files from SciFinder and then using ChemDraw Professional (ver. 20.1.0.110). All SMILES were converted to canonical SMILES using OpenBabelGUI (ver. 3.1.1 ,O'Boyle et al., 2011). In total, 429 triterpenes with activity test results were obtained. The SMILES of collected triterpenes and references were shown in Supplementary Material S1.

The activity values were expressed as IC_50,_ EC_50_, and inhibition rate. The activity expressed in *μ*g/mL was transformed into *μ*M using the following equation.

Activity value(μM)=activity value(μg/mL)/molecular weight×1000

We set the threshold at 25 *μ*M to define anti-HSV-1 active/inactive. Data with activity expressed as inhibition rates were judged to be active or inactive, considering the test concentration. If the test concentration was smaller than 25 *μ*M and the inhibition rate did not exceed 50%, the data were excluded because the exact activity value could not be determined. Several activity data from the ChEMBL database were represented as “active” or “inactive.” This category was applied to the classification as it stands. For compounds with multiple activity test results, the median value was derived and assigned to a class.

### Molecular Representation

Molecular descriptors were used to convert the chemical structures into numerical values. Molecular descriptors are features created based on the physical properties and partial structures of compounds (Brown, 2018). The mol files of each compound were downloaded from PubChem (Kim et al., 2021) or constructed from CAS numbers or canonical SMILES using ChemDraw Professional. The structures of all triterpenes were carefully checked for their conformations. Then, each compound was converted to 3874 descriptors using mol files using alvaDesc (ver. 1.0.16). Variable reduction was conducted to eliminate any values inappropriate for model building in the following four steps: (1) eliminate descriptors with one or more missing values, (2) eliminate descriptors with all constant values or near constant values, (3) eliminate descriptors with standard deviations less than 0.01, and (4) eliminated one of the descriptors if the pair correlation coefficient was larger or equal to 0.75. After variable reduction, 267 descriptors were standardized and used to build a predictive model. The number of descriptors for each block is listed in Table 1.

**TABLE 1:** Number of the molecular descriptors using model construction.

Descriptors	Number of descriptors
2D Atom Pairs	48
2D autocorrelations	37
2D matrix-based descriptors	34
Atom-centered fragments	22
Atom-type E-state indices	10
Burden eigenvalues	7
Constitutional indices	7
Edge adjacency indices	15
ETA indices	4
Functional group counts	21
Information indices	4
P_VSA-like descriptors	12
Pharmacophore descriptors	31
Ring descriptors	9
Topological indices	4
Walk and path counts	2

### Principal Component Analysis

Principal component analysis (PCA) was performed to check the distribution of the compounds from ChEMBL, DNP, and original papers. The 267 structural descriptors, which are the same as those used for constructing the predictive model, were used as variables in the PCA. All compounds were visualized in three-dimensional space. PCA and visualization were performed using JMP® Pro (ver. 15.1.0).

### Model Construction

Prior to the construction of the predictive model, we developed initial models using various prediction methods to determine which methods were suitable for predicting anti-HSV-1 activity. Initial models were constructed with logistic regression, random forest, decision tree, support vector machine, and k-nearest neighbor methods by fivefold cross-validation (data not shown). Among the initial models, the model using logistic regression showed the best performance. Therefore, we used the logistic regression (LR) algorithm to construct the classification model. The parameters of the model were automatically optimized using the grid search algorithm. The division of the data into training and testing sets was carried out at a ratio of 4:1. We performed 10 attempts with different divisions methods and employed the optimal split. All the steps of model construction were implemented in Python (ver. 3.7.3) using the scikit-learn (ver. 0.20.3) library, which is a machine learning package.

The LR algorithm is the major classification method that is mainly applied to linearly separable problems. In this method, the probability of an active or inactive class is obtained by utilizing a logistic function. Three parameters of the LR classification model were optimized by grid search CV to improve the model performance: solver = {liblinear, saga, newton-cg, sag, lbfgs}, penalty = {l1, l2}, and C = {0.1, 1, 10, 100}.

### Evaluation of the Model Performance

The performance of the constructed model for predicting anti-HSV-1 activity was evaluated in terms of accuracy, sensitivity, precision, F1-score, and receiver operating characteristic (ROC)—area under the curve (AUC) by fivefold cross-validation. The definitions of these metrics are as follows:

$$\mathrm{Accuracy}=\frac{\mathrm{TP}+\mathrm{TN}}{\mathrm{TP}+\mathrm{TN}+\mathrm{FP}+\mathrm{FN}}$$

$$\mathrm{Sensitivity}=\frac{\mathrm{TP}}{\mathrm{TP}+\mathrm{FN}}$$

$$\mathrm{Precision}=\frac{\mathrm{TP}}{\mathrm{TP}+\mathrm{FP}}$$

$$F1\;\mathrm{score}=\frac{2\times sensitivity\times precision}{sensitivity+precision}$$

where TP denotes true positive, TN denotes true negative, FP denotes false positive, and FN denotes false negative. ROC is a graph plotting FPR and TPR with threshold change, and its AUC is the evaluation index of the model. The values of ROC-AUC were closer to 1, which indicates a higher model performance.

The QSAR model encounters some difficulty in constructing accurate predictions for compounds that are dissimilar to the structure of the compound used for training. Therefore, the range of predictable compounds should be defined as the applicability domain (AD) (OECD, 2014). The compounds were checked for similarity to the training compounds using a one-class support vector machine (OCSVM) (Kaneko et al., 2015), and the compounds in the AD were used for prediction and evaluation. To perform the OCSVM method, all compounds were represented by extended connectivity fingerprints (ECFP4).

### Compounds for the anti-HSV-1 Assay

The 20 test triterpenes for the HSV-1 assay were provided by a natural product library assembled by the Department of Pharmacognosy at Kyoto Pharmaceutical University (Figure 2). The triterpenes were isolated from three plants, including *I. japonicus*, *P. glomerata*, and *I. obliquus*. Euscaphic acid **(1)**, bayogenin **(2)**, 2α,3α,23-trihydroxyurs-12-en-28-oic acid **(3)** were isolated from the aerial parts of I. japonicus. Pfaffiaglycoside B **(4)**, (20α)-3-oxoolean-12-ene-28,29-dioic acid **(5)**, pfaffianol A **(6)**, pterosterone **(7)**, taxisterone **(8)**, 2β,3β,14α,17β-tetrahydroxy-5β-androst-7-en-6-one **(9)**, oleanolic acid 3-O-β-D-glucuronopyranoside **(10)**, chikusetsusaponin IVa **(11)**, boussingoside A2 **(12)**, pfaffoside C **(13)**, oleanolic acid 28-O-β-D-glucopyranoside **(14)**, 22-oxo-20-hydroxyecdysone **(15)** were isolated from the roots of P. glomerata. 5α,8α-epidioxyergosta-3β-ol **(16)**, 3β,22*R*-Dihydroxylanosta-8,23*E*-diene-25-peroxide **(17)**, 3β-hydroxylanosta-8,24-dien-21-al **(18)**, lanosterol **(19)**, and (3β,23E)-25-hydroperoxylanosta-8,23-dien-3-ol **(20)** were isolated from the sclerotia of I. obliquus. The isolation procedure and structural determination of these compounds have been described in previous reports (Nakamura et al., 2009; Nakamura et al., 2010).

### Evaluation of Anti-HSV-1 Activity and Cytotoxicity

Antiviral effects against HSV-1 (HF strain: acyclovir-sensitive) were evaluated using a plaque reduction assay with some modifications as previously reported (Ogawa et al., 2018). The activity of all test compounds was measured at 25 *μ*M, corresponding to the threshold of the prediction model. Compounds with significantly strong activity at 25 *μ*M were further measured at lower concentrations to calculate the IC_50_. The protocol was performed follows.

Vero cells were maintained in Dulbecco’s Modified Eagle Medium (DMEM) supplemented with 10% fetal calf serum (FBS). Vero cells (1.7–2.8 × 10^5^ cells/well) were seeded onto a 12-well tissue culture plate and precultured for 1–2 days. The cells were inoculated with 100 plaque-forming units (PFU) of HSV-1 in 0.1 ml of FBS-free DMEM. After 30 min, the inoculum was removed. HSV-infected cells were maintained in DMEM containing 10% FBS, methylcellulose, and serially diluted test compounds. After 48 h of incubation at 37°C, the medium was removed. The cell sheets were stained with 1% crystal violet dissolved in 50% methanol, and the number of plaques larger than 0.4 mm was counted. HSV-1 replication causes cytopathic effects, resulting in plaque formation. The number and size of plaques reflected virus yields. To examine the effect of virus yield reduction, the total plaque number in cells untreated with the compound was defined as 100% and set as the control group. The significant difference of test compounds at 25 *μ*M from the control group was calculated using the Student’s *t*-test. The significant difference of each concentration of **10**–**13** and **16** from the control group was calculated by Dunnett’s test. Probability (*p*) values less than 0.05 were considered to be significant (^*^ *p* < 0.05, ^**^ *p* < 0.01).

All test compounds were evaluated their cytotoxicity by using CellTiter-Glo 3D® cell viability assay (Matsumoto et al., 2020). Briefly, Vero cells (3.0 × 10^3^ cells/ well) were seeded in 96-well plates (96F Nunclon TM Delta Microwell SI; Thermo Fisher Scientific), and precultured for 24 h. The cells were treated with test compounds (10 *μ*L/well). After 48 h, CellTiter-Glo® 3D Reagent (Promega, Madison, WI, United States) was added at 100 *μ*L per well, mixed by shaking for 5 min at RT, and incubated for 25 min at 37°C. The cell-containing media were transferred to a 96-well white plate (96F Nunclon TM Delta White Microwell SI; Thermo Fisher Scientific). Luminescence was measured with a luminometer (FLUOstar OPTIMA®; BMG Labtech, Ortenberg, Germany).

To verify the performance of the constructed model, 20 compounds were predicted to be active or inactive and checked with the assay results. Compounds within the AD defined by OCSVM were predicted for their anti-HSV-1 activity.

## Results

### Overview of the Collected Data

**FIGURE 1:**
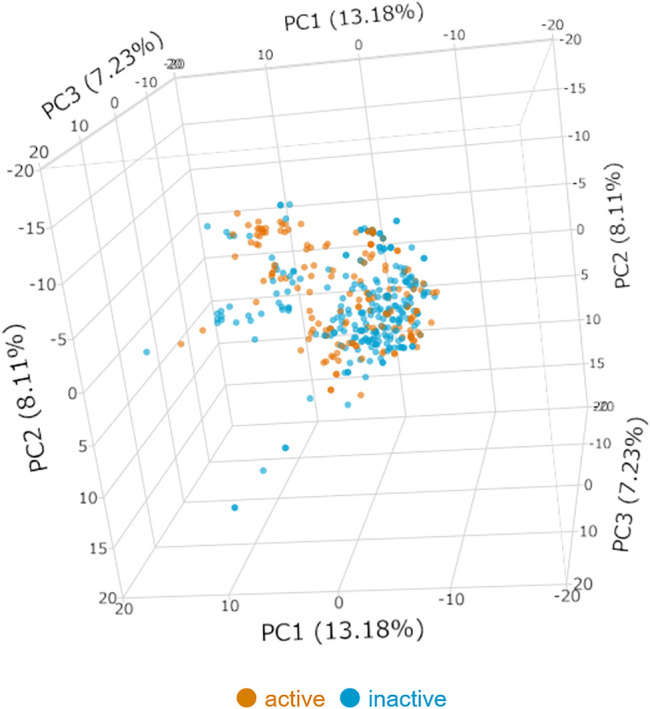
Principal component analysis of the active or inactive compounds of the training dataset. PC = principal component. Blue dot: HSV-1 inactive, orange dot: HSV-1 active.

For the data collection, 472 triterpenes were collected from ChEMBL, DNP, and original papers. After data cleaning and Setting threshold, 416 triterpenes with activity data were used as training and evaluation. The anti-HSV-1 assay conditions for these data included incubation times of 12–48 h and cell types of HeLa cells, Vero cells, and virus strains of mainly KOS (ATCC^®^ VR1493), Strain F (ATCC^®^ VR-733), and 17 (GenBank acc. no. NC_001,806) and HSV-1 MI (ATCC^®^ VR-539), which were acyclovir-sensitive strain. With the threshold of activity set to 25 *μ*M, 166 active and 250 inactive compounds were defined (Table 2); Figure 1 shows the results of the PCA of active or inactive compounds collected from ChEMBL, DNP, and the original papers using 267 descriptors. The first three principal components (PC), namely, PC-1, PC-2, and PC-3, showed 13.18, 8.11, and 7.23% variance, respectively. The total variance of the first 3 PC was 28.52%. The distribution of anti-HSV-1 active compounds and inactive compounds seemed to be similar, whereas they were slightly wider in the inactive compounds.

**TABLE 2:** Number of compounds for each dataset.

	Train	Test	External validation
Number of compounds	332	84	20
active/inactive	128/204	38/46	2/18
Number of compounds inside AD	‒	71	18
active/inactive	‒	31/40	2/16

AD, applicability domain.

**FIGURE 2:**
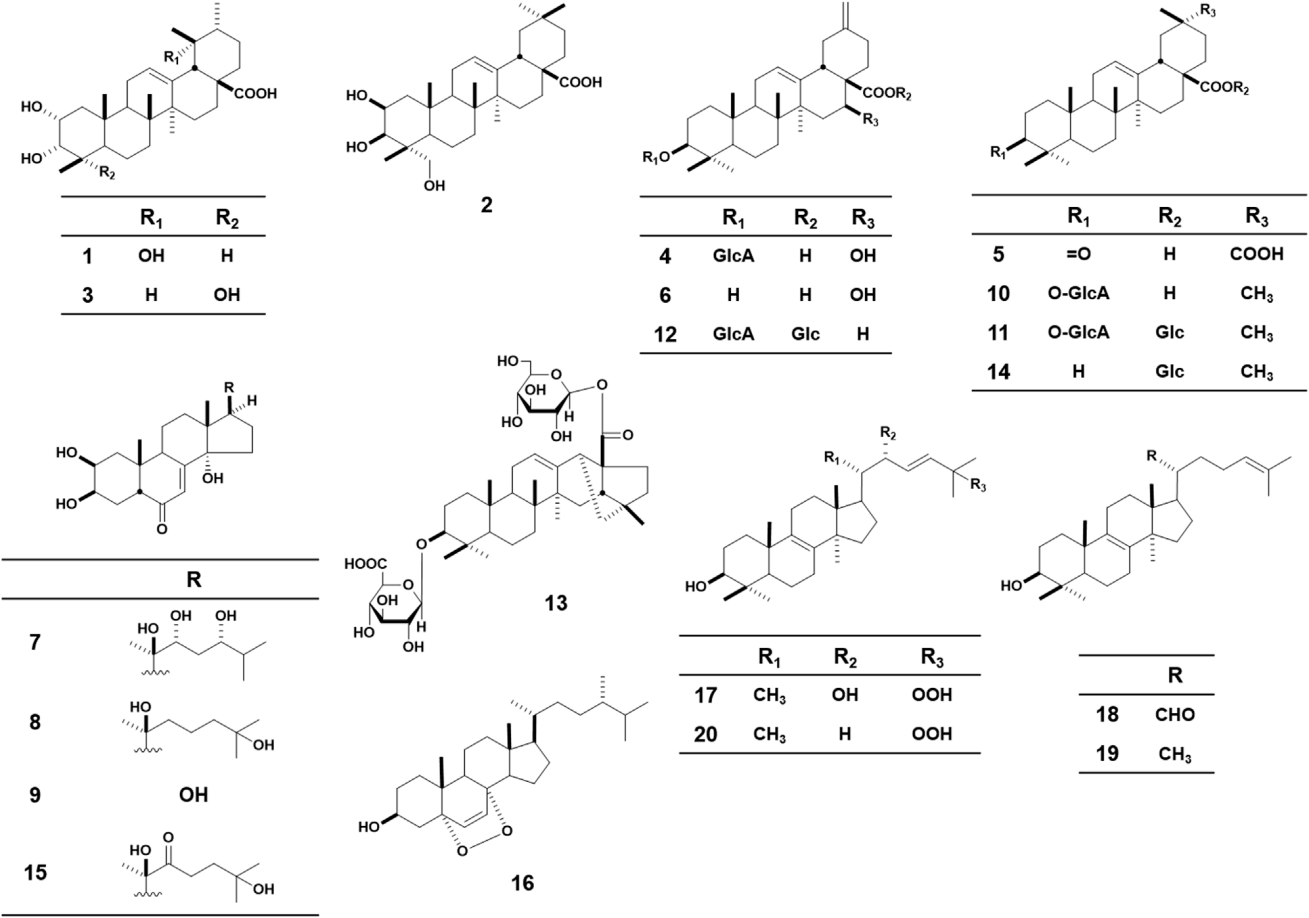
Chemical structures of 20 triterpenes.

### Predictive Model for the Anti HSV-1 Effect of Triterpenes

A predictive model for anti-HSV-1 activity was constructed using collected triterpene compounds. The structures of the triterpenes were initially converted to 3874 2D molecular descriptors. In this study, the targeted compounds were confined to triterpenes; hence, some of the structural descriptors had similar values. Therefore, in order to select structural descriptors that clearly identified compounds, descriptor reduction was performed with a correlation coefficient of 0.75 and other conditions. Consequently, 267 descriptors were used to construct a predictive model with classification using logistic regression. Optimization using 5-fold cross-validation showed that the best parameters involved setting the solver = “liblinear”, C = 1.0, and penalty = “l2”. At these optimal parameter settings, the accuracy was 0.77, AUC was 0.86, sensitivity was 0.71, precision was 0.77 and F1-score was 0.74 (Table 3).

**TABLE 3:** Performance of the predictive model for the anti-HSV-1 activity.

	**Accuracy**	**Sensitivity**	**Precision**	**F1 score**	**ROC-AUC**
Grid search	0.77	0.71	0.77	0.74	0.86
Test	0.79	0.76	0.76	0.76	0.86
Inside AD	0.78	0.71	0.76	0.73	0.87

AD, Applicability domain; ROC-AUC, receiver operating characteristic—area under the curve.

Grid search, Results of model optimization using AUC as an indicator; Test, Prediction results of the test data without considering AD using the optimized model; Inside AD, Prediction results of test data considering AD using the optimized model.

The range of compounds that can be applied to the prediction model depends on the structural diversity of the compounds used for training. Thus, we considered AD for this predictive model from the perspective of chemical space. In this study, the AD was determined using the OCSVM method. Among 84 test compounds, 13 of the test compounds were excluded from the prediction because they were judged to be out of the AD. For the remaining 71 test compounds determined to be within the AD, the optimized predictive model was used to predict anti-HSV-1 active/inactive, with an accuracy of 0.78 and an AUC of 0.87. On the other hand, without considering AD, the accuracy was 0.79 and the AUC was 0.86.

### Evaluation of the anti-HSV-1 Effect of Triterpenes and Prediction Results

A plaque reduction assay and the cytotoxicity assay were performed to evaluate the 20 triterpenes. The anti-HSV-1 activity of all triterpenes was evaluated at 25 *μ*M, and compounds which showed significant anti-HSV-1 activity at 25 *μ*M were further evaluated at 5 and 10 *μ*M. The result of the cytotoxicity assay was described in Supplementary Material S1. The therapeutic agent acyclovir (IC_50_: 3.08 *μ*M, cell viability: 102.92 ± 13.78%, at 10 *μ*M) and the typical triterpene oleanolic acid (inhibition: 21.40 ± 11.56% at 25 *μ*M) were used as positive controls. As a result, 4 triterpenes **10** (83.33 ± 41.11%), **11** (51.83 ± 9.61%), **12** (24.72 ± 10.22%), **13** (27.19 ± 5.52%), showed significant anti-HSV-1 activity at 25 *μ*M (Table 4). In particular, **11** exhibited potent activity with IC_50_ of 13.06 *μ*M respectively with no cytotoxicity (viability: 111.53 ± 4.45%). Compound **10** showed 14.13 *μ*M of IC_50_, however cytotoxicity was observed at 25 *μ*M (viability: 14.64 ± 2.88%). At 10 *μ*M, **10** showed significant anti-HSV-1 (27.49 ± 8.92%) activity with no cytotoxicity (viability: 103.99 ± 9.28%). Compounds **10** and **11** were both oleanolic glycosides with a glucuronic acid at the 3-position, and **11** also had glucose at the 28-position.

**TABLE 4:** Anti-HSV-1 activity of triterpenes.

Compound	Origin	Inhibitory rate (%)^a^			IC_50_ (*μ*M)	Prediction result	Probability
		5 *μ*M	10 *μ*M	25 *μ*M			
1	*Isodon japonicus*	**‒**	**‒**	0.00 ± 11.89	**‒**	inactive	0.8848
2		**‒**	**‒**	‒2.99 ± 8.99	**‒**	active	0.5265
3		**‒**	**‒**	‒4.82 ± 12.38	**‒**	inactive	0.9522
4	*Pfaffia glomerata*	**‒**	**‒**	7.64 ± 10.72	**‒**	inactive	0.7321
5		**‒**	**‒**	‒4.36 ± 23.98	**‒**	active	0.8738
6		**‒**	**‒**	12.39 ± 16.94	**‒**	inactive	0.5545
7		**‒**	**‒**	‒29.00 ± 6.81^*^	**‒**	inactive	0.9923
8		**‒**	**‒**	‒21.60 ± 11.92	**‒**	inactive	0.8345
9		**‒**	**‒**	‒2.48 ± 6.80	**‒**	‒^b^	‒^b^
10		5.85 ± 8.40	27.49 ± 8.92^**^	83.33 ± 41.11^**,^ ^c^	14.13	active	0.6441
11		14.56 ± 15.02	36.83 ± 17.39^**^	51.82 ± 9.61^**^	13.06	active	0.8005
12		4.42 ± 8.12	2.77 ± 11.62	24.72 ± 10.22^*^	**‒**	active	0.9833
13		9.52 ± 11.74	2.38 ± 17.69	27.55 ± 11.00	**‒**	‒^b^	‒^b^
14		**‒**	**‒**	10.09 ± 20.33	**‒**	inactive	0.7969
15		**‒**	**‒**	‒16.03 ± 8.69	**‒**	inactive	0.6087
16	*Inonotus obliquus*	−2.49 ± 8.74	0.00 ± 12.87	27.19 ± 5.52^**^	**‒**	inactive	0.7212
17		**‒**	**‒**	‒3.67 ± 1.53^d^	**‒**	inactive	0.9756
18		**‒**	**‒**	1.24 ± 3.80	**‒**	active	0.9791
19		**‒**	**‒**	20.42 ± 8.49	**‒**	inactive	0.6141
20		**‒**	**‒**	19.03 ± 9.25	**‒**	inactive	0.9740
		**conc.**	**5 *μ*M**	**25 *μ*M**			
oleanolic acid	positive control		9.02 ± 4.22	21.40 ± 11.56^*^	**‒**	‒	‒
		**conc.**	**2.5 *μ*M**	**10 *μ*M**			
acyclovir	positive control		57.51 ± 11.88^**^	96.01 ± 11.76^**^	3.08	‒	‒

IC_50_ = 50% inhibitory concentration.

a Significantly different from the control group, ^*^ *p* < 0.05, **p < 0.01.

b Compounds **9** and **13** were judged to be outside the applicability domain.

c The cell viability of compound **10** at 25 μM was 14.64 ± 2.88%. Compound **10** did not show the cytotoxicity at 10 μM (104.0 ± 9.28%).

d The cell viability of compound **17** at 25 μM was 16.84 ± 9.50%.

The anti-HSV-1 activity of these 20 compounds was also predicted using an optimized model. The OCSVM was used to determine whether these triterpenes were within the AD of the model, and 18 of the 20 compounds were acceptable for this model. Compounds **9** and **13** were judged to be outside the AD; thus, they were excluded from the compounds to be predicted. As a result of prediction, 6 triterpenes (**2**, **5**, **10**, **11**, **12** and **18**) were determined to be active and **12** triterpenes (**1**, **3**, **4**, **6**–**8**, **14**–**17** and **19**–**20**) were determined to be inactive.

Then, the results of the anti-HSV-1 assay and prediction were compared. Compounds **11**, which showed anti-HSV-1 activity stronger than 25 *μ*M of IC_50_, were predicted active with high probability (**11**: 0.8005). Overall, the accuracy and AUC of our model for these external-validation compounds were 0.83 and 0.81, respectively (Table 5). The sensitivity was 1.00, and the F1-score was 0.40.

**TABLE 5:** Prediction performance for 20 triterpenes.

Accuracy	Sensitivity	Precision	F1 score	ROC-AUC
0.83	1.00	0.40	0.57	0.81

ROC-AUC, receiver operating characteristic—area under the curve.

## Discussion

The chemical structure of natural products is a rich source of medicinal seeds (Newman and Cragg, 2020). However, many efforts have been made to find compounds with the desired activity. The development of a new approach to efficiently determine the desired activity was therefore required.

There has been a need for anti-HSV-1 therapeutic agents with a different skeleton from that of the existing nucleobases. Natural products are good source to develop the efficient and safe treatment of HSV-1 infections (Treml et al., 2020). There have been various types of anti-HSV-1 active natural products such as terpenoids, flavonoids, polysaccharides and other miscellaneous compounds. Among them, terpenes showed the highest percentage (34.4%) of the reported natural products with anti-HSV-1 activity (Zhong et al., 2013). In particular, they mentioned that glycosides are useful as active compounds. However, most SAR remain unclear.

In recent years, QSAR research has been actively conducted. For instance, Masand et al. constructed a QSAR model for SARS-CoV inhibitors from a dataset of peptide compounds (Masand et al., 2020). Banerjee et al. reported on the development of a predictor that used structural descriptors to classify the taste of compounds (Banerjee et al., 2018). As these reports show, QSAR studies have successfully provided new insights into SAR and new possibilities for the discovery of active compounds. For anti-HSV-1 activity, Sabatino et al. reported a predictive model for essential oils that exhibit antiviral activity using the partial least square discriminant analysis algorithm (Sabatino et al., 2020). Another report described the protein‒protein interaction between human and HSV-1 using machine learning techniques (Lian et al., 2020). However, to the best of our knowledge, predictive models for compounds that exhibit anti-HSV-1 activity have not been investigated so far.

Considering these backgrounds, we have started this study to accomplish a comprehensive understanding by integrating the information from previous original papers with machine learning techniques. Briefly, we constructed a predictive model for anti-HSV-1 activity by summarizing previous activity reports on triterpenes to efficiently identify the active triterpenes. Despite the diversity of the assay condition used for training, such as incubation time, cell types, and virus strains, our model was successfully developed with high predictive performance. On the other hand, there is still a need for further information on the anti-HSV-1 activity of triterpenes. We evaluated the anti-HSV-1 activity of 20 triterpenes using a plaque reduction assay and identified several active compounds.

In the step of collecting information on the anti-HSV-1 activity of triterpenes, the activity information of triterpenes, saponins, and steroids with different skeletons were obtained. We set the activity threshold to 25 *μ*M IC_50_. The reason for this threshold is that empirically accessible to obtain compounds that do not exhibit cytotoxicity but exhibit anti-HSV-1 activity. Additionally, sufficient active and inactive data were available to construct the model, and the active and inactive compounds were evenly divided in terms of their structures. PCA analysis was performed using 267 molecular descriptors, as in the model construction process. This result showed that the distributions of the active and inactive compounds were similar. The results of model construction indicated that our predictive model showed high discriminant ability with an accuracy of 0.79, and an AUC of 0.86, even though the training data were based on non-unified assay protocols. The precision and sensitivity were 0.76, and 0.76, respectively, suggesting that our predictive model is effective in finding active compounds with a good balance. In addition, it should be emphasized that our model showed high discrimination ability, not only for simple triterpenes but also for their glycosides and steroids.

In a prediction model using compound structures, determining whether the compound to be predicted is in the AD of the model based on structural similarity is generally necessary. In this study, the OCSVM method was used to define the AD. Consequently, 13 out of 84 test compounds were found to be outside the AD. Among the 13 excluded compounds, 7 were active and 6 were inactive. When comparing the prediction performance of using and not considering AD, we found that the accuracy was slightly higher when AD was not used than when using AD. In this study, we limited the targets to triterpene skeletons; thus, the structural similarity was considered initially high.

In the anti-HSV-1 assay, four triterpenes (**10**, **11**, **12**, and **16**) exhibited significant activity against HSV-1. Specifically, compounds **11** showed a potent anti-HSV-1 activity with IC_50_ of 13.06 *μ*M. Compound **11** was previously reported to exhibit anti-HSV-1 activity (Rattanathongkom et al., 2009). This report was not included in the training data because it did not include our search words. Our result on the activity of **11** was also supportive of a previous study. In our classidication model, which was created by integrating various protocols, compound **11** was predicted to have a high probability (0.8005) of showing activity. This suggests that compound **11** may be broadly active under different assay conditions, such as different cell types and virus strains. compound **10** showed IC_50_ of 14.13 *μ*M. Although significant cytotoxicity was observed at 25 *μ*M, no cytotoxicity was observed at 10 *μ*M. It indicated that **10** can be treated as an active compound, with a narrow safety range. Previously, Rattanathongkom et al. reported that **10** did not show any anti-HSV-1 activity but cytotoxicity. Our classification model classified compound **10** as being in the active group with a probability of 0.6441, consistent with our results. Because the probability was near the threshold (0.5), the classification of **10** as active or inactive was sensitive. However, it can be regarded as a good reflection of the current and previous results. From these results, it is meaningful to check the probability of the predictive model proposed in this study to examine the certainty of active compounds.

The active compounds included only three oleanane (**10**, **11**) or noroleanane-type (**12**) saponins with glucuronic acid at the hydroxy group at the 3-position and an ergostene-type triterpene with a peroxide group. Compounds **10** and **11** showed potent anti-HSV-1 activity with an IC_50_ of less than 25 *μ*M. In addition compound **12** exhibited significant activity and compound **13** showed no significant activity, glucuronic acid at position 3 may have been involved in the expression of the anti-HSV-1 activity. Compound **10** showed cytotoxicity, while **11** and **12** with glucose at position 28 showed no cytotoxicity, suggesting that glucose at position 28 may be involved in reducing cytotoxicity. All the compounds that showed activity had a hydroxy group at the 3-position and no substituent at the 2-position. On the other hand, compounds with hydroxy groups at both 2 and 3 positions showed no activity. This suggests that the hydroxy group at the 2-position may be involved in the decrease in activity. Comparing the activity of compounds with or without glucose at position 28, the compounds with glucose seemed to be slightly more active. However, compound **14**, which had glucose at position 28, but no glucuronic acid at position 3, did not show significant activity. Therefore, glucose at position 28 may have less of an effect on activity than glucuronic acid at position 3.

When comparing the results from the constructed predictive model and the results from the anti-HSV-1 assay, the predictive model successfully found both **10** and **11** as active compounds, which showed IC_50_ stronger than 25 *μ*M. The predictions for 20 compounds showed high sensitivity (1.00), but lower precision (0.40). This predictive model is expected to improve the efficiency of finding the active compounds. Therefore, it is more important not to miss the potentially active compounds (sensitivity) than to find them accurately (precision). Meanwhile, compound **12** was determined to be active with a high probability (0.9833), but the prediction was incorrect. However, **12** showed a moderate anti-HSV-1 activity at 25 *μ*M, suggesting that the prediction model can play a role in identifying compounds with potential anti-HSV-1 activity. Considering these facts, the prediction model was consistent with our purpose.

## Conclusion

This study was conducted to improve the search efficiency for active triterpenes. We have successfully developed a predictive model for anti-HSV-1 activity by integrating the results of previous reports. The predictive model showed satisfactory performance with an accuracy of 0.79, AUC of 0.86, and high ability to identify active compounds. In addition, we measured the anti-HSV-1 activity of 20 triterpenes from a natural product library. We found that several triterpenes showed a significant anti-HSV-1 activity at 25 *μ*M. Among them, **11** showed significantly strong activity, with IC_50_ values of 13.06 *μ*M with no toxicity. Further, our prediction model showed high prediction performance with an accuracy of 0.83 and AUC of 0.81, even in external validation using these triterpenes in assay. The proposed model succeeded in finding all compounds that showed stronger activity than 25 *μ*M of IC_50_. This indicates that the model can determine the anti-HSV-1 activity of various structural triterpenes, their glycosides, and sterols with good performance.

In this study, our binary classification model was able to integrate the results of assays with different experimental protocols and filter out active compounds based on a comprehensive relationship pattern. Using this predictive model, it is possible to determine which triterpenes are likely to be active, allowing for more rapid access to anti-HSV-1 active triterpenes. However, because the training for the prediction model depends on known data, there are some limitations in this approach. It is difficult to accurately determining the activity of a rare compound because the training data would not contain structurally similar compounds to rare compounds. Therefore, it is expected that further improvement of the predictive model will be achieved by accumulating more data on the anti-HSV-1 activity of various compounds.

## Data Availability

The original contributions presented in the study are included in the article/Supplementary Material, further inquiries can be directed to the corresponding authors.
