# Supplementary material for: Effective Search of Triterpenes with Anti-HSV-1 Activity Using a Classification Model by Logistic Regression
Source: Front Chem. 2021 Nov 2;9:763794. doi: 10.3389/fchem.2021.763794 (PMC8593400; doi:10.3389/fchem.2021.763794)
Supplement: Supplementary file 1 [file DataSheet1.pdf]

Supplementary Material

An Effective Search of Triterpenes with Anti-HSV-1 Activity Using Classification Model by Logistic Regression

Keiko Ogawa[1*], Seikou Nakamura[2*], Haruka Oguri[1], Kaori Ryu[2], Taichi Yoneda[2], Rumiko Hosoki[1]

[1]Laboratory of Regulatory Science, College of Pharmaceutical Sciences, Ritsumeikan University, Kusatsu, Japan

[2]Department of Pharmacognosy, Kyoto Pharmaceutical University, Kyoto, Japan

S1. The canonical SMILES of collected triterpene with information of anti-HSV-1 activity and their references.

S2. The result of cytotoxicity assay

S3. References

S. 1. The canonical SMILES of collected triterpene with information of anti-HSV-1 activity and their references.

CC[C@@H](C(C)C)/C=C/[C@H]([C@H]1CC[C@@H]2[C@]1(C)CC[C@H]1[C@H]2CC=C2[C@]1(C)CC[C@@H](C2)O)C	
CC[C@@H]([C@H]([C@@H]([C@H]([C@H]1CC[C@@H]2[C@]1(C)CC[C@H]1[C@H]2CC(=O)[C@@H]2[C@]1(C)C[C@@H](O)[C@H](C2)O)C)O)O)C(C)C	
CC[C@H]([C@H]([C@@H]([C@H]([C@H]1CC[C@@H]2[C@]1(C)CC[C@H]1[C@H]2CC(=O)[C@@H]2[C@]1(C)CC[C@@H](C2)OC(=O)C)C)O)O)C(C)C	
CC[C@H]([C@@H]([C@H]([C@H]([C@H]1CC[C@@H]2[C@]1(C)CC[C@H]1[C@H]2CC(=O)[C@@H]2[C@]1(C)CC[C@@H](C2)OC(=O)C)C)O)O)C(C)C	
CC[C@H]([C@H]([C@@H]([C@H]([C@H]1CC[C@@H]2[C@]1(C)CC[C@H]1[C@H]2CC(=O)[C@@H]2[C@]1(C)CC[C@@H](C2)Br)C)O)O)C(C)C	
CC[C@H]([C@@H]([C@H]([C@H]([C@H]1CC[C@@H]2[C@]1(C)CC[C@H]1[C@H]2CC(=O)[C@@H]2[C@]1(C)CC[C@@H](C2)Br)C)O)O)C(C)C	
CC[C@H]([C@H]([C@@H]([C@H]([C@H]1CC[C@@H]2[C@]1(C)CC[C@H]1[C@H]2CC(=O)[C@@]2([C@]1(C)CC[C@@H](C2)O)O)C)O)O)C(C)C	
CC[C@H]([C@@H]([C@H]([C@H]([C@H]1CC[C@@H]2[C@]1(C)CC[C@H]1[C@H]2CC(=O)[C@@]2([C@]1(C)CC[C@@H](C2)O)O)C)O)O)C(C)C	
CC[C@H](C(C)C)/C=C/[C@H]([C@H]1CC[C@@H]2[C@]1(C)CC[C@H]1[C@H]2CC(=O)[C@@]2([C@]1(C)CC[C@@H](C2)O)O)C	
CC[C@H]([C@H]([C@@H]([C@H]([C@H]1CC[C@@H]2[C@]1(C)CC[C@H]1[C@H]2COC(=O)[C@@H]2[C@]1(C)C[C@@H](O)[C@H](C2)O)C)O)O)C(C)C	
CC[C@@H](C(C)C)/C=C/[C@H]([C@H]1CC[C@@H]2[C@]1(C)CC[C@H]1[C@H]2CC(=O)[C@@]2([C@]1(C)CC=CC2)F)C	
CC[C@@H](C(C)C)/C=C/[C@H]([C@H]1CC[C@@H]2[C@]1(C)CC[C@H]1[C@H]2CC(=O)[C@@]2([C@]1(C)CC[C@@H](C2)F)Cl)C	
CC[C@H]([C@H]([C@@H]([C@H]([C@H]1CC[C@@H]2[C@]1(C)CC[C@H]1[C@H]2CC(=O)[C@@]2([C@]1(C)CC[C@@H](C2)O)F)C)O)O)C(C)C	
CC[C@H]([C@@H]([C@H]([C@H]([C@H]1CC[C@@H]2[C@]1(C)CC[C@H]1[C@H]2CC(=O)[C@@]2([C@]1(C)CC[C@@H](C2)O)F)C)O)O)C(C)C	(Wachsman et al. 2004)
CC[C@H]([C@H]([C@@H]([C@H]([C@H]1CC[C@@H]2[C@]1(C)CC[C@H]1[C@H]2CC(=O)[C@@H]2[C@]1(C)CC[C@@H](C2)F)C)O)O)C(C)C	
CC[C@H]([C@@H]([C@H]([C@H]([C@H]1CC[C@@H]2[C@]1(C)CC[C@H]1[C@H]2CC(=O)[C@@H]2[C@]1(C)CC[C@@H](C2)F)C)O)O)C(C)C	
CC[C@H]([C@H]([C@@H]([C@H]([C@H]1CC[C@@H]2[C@]1(C)CC[C@H]1[C@H]2CC(=O)[C@@]2([C@]1(C)CC[C@@H](C2)F)O)C)O)O)C(C)C	
CC[C@H]([C@@H]([C@H]([C@H]([C@H]1CC[C@@H]2[C@]1(C)CC[C@H]1[C@H]2CC(=O)[C@@]2([C@]1(C)CC[C@@H](C2)F)O)C)O)O)C(C)C	
CC[C@H]([C@H]([C@@H]([C@H]([C@H]1CC[C@@H]2[C@]1(C)CC[C@H]1[C@H]2CC(=O)[C@@H]2[C@]1(C)CC[C@H](C2)F)C)O)O)C(C)C	
CC[C@@H](C(C)C)/C=C/[C@H]([C@H]1CC[C@@H]2[C@]1(C)CC[C@H]1[C@H]2C[C@H]([C@@]2([C@]1(C)CC[C@@H](C2)Br)Cl)O)C	
CC[C@@H](C(C)C)/C=C/[C@H]([C@H]1CC[C@@H]2[C@]1(C)CC[C@H]1[C@H]2CCC2=CC(=O)CC[C@]12C)C	
CC[C@@H](C(C)C)/C=C/[C@H]([C@H]1CC[C@@H]2[C@]1(C)CC[C@H]1[C@H]2C[C@@H]([C@@H]2[C@]1(C)C[C@@H](O)[C@H](C2)O)F)C	
CC[C@H]([C@H]([C@@H]([C@H]([C@H]1CC[C@@H]2[C@]1(C)CC[C@H]1[C@H]2CC(=O)[C@@H]2[C@]1(C)CC[C@H](C2)O)C)O)O)C(C)C	
CC([C@@H]([C@H]1OC(O[C@@H]1[C@H]([C@H]1CC[C@@H]2[C@]1(C)CC[C@H]1[C@H]2CC(=O)[C@@H]2[C@]1(C)C[C@H]1OC(O[C@H]1C2)(C)C)C)(C)C)C)C	
CC[C@H]([C@@H]([C@H]([C@H]([C@H]1CC[C@@H]2[C@]1(C)CC[C@H]1[C@H]2CC(=O)[C@@]2([C@]1(C)C[C@@H](O)[C@H](C2)O)F)C)O)O)C(C)C	
CC[C@H]([C@H]([C@@H]([C@H]([C@H]1CC[C@@H]2[C@]1(C)CC[C@H]1[C@H]2CC(=O)[C@@H]2[C@]1(C)CC[C@@H](C2)O)C)O)O)C(C)C	
CC[C@H]([C@@H]([C@H]([C@H]([C@H]1CC[C@@H]2[C@]1(C)CC[C@H]1[C@H]2CC(=O)[C@@]2([C@]1(C)CC[C@H](C2)O)O)C)O)O)C(C)C	
CC[C@H]([C@H]([C@@H]([C@H]([C@H]1CC[C@@H]2[C@]1(C)CC[C@H]1[C@H]2CCC2=CC(=O)CC[C@]12C)C)O)O)C(C)C	

CC[C@H]([C@@H]([C@H]([C@H]([C@H]1CC[C@@H]2[C@]1(C)CC[C@H]1[C@H]2CCC2=CC(=O)CC[C@]12C)C)O)O)C(C)C	
OCC1O[C@@H](OC2[C@@H](OC[C@H]([C@H]2O)C)O[C@H]2CC[C@]3([C@H]([C@]2(C)CO)CC[C@@]2([C@@H]3CC[C@]34[C@@]2(C)C[C@H]([C@@]2([C@H]4CC(C)(C)CC2)CO3)O)C)C)C([C@@H]([C@@H]1O[C@@H]1OC[C@@H]([C@H](C1O)O)O)O[C@@H]1OC(CO)[C@H]([C@H](C1O)O)O)C	(Amoros et al. 1987)
OC[C@H]1O[C@@H](OC(=O)[C@@]23CC[C@H]([C@@]([C@H]3C3=CC[C@H]4[C@@]([C@@]3(CC2)C)(C)CC[C@@H]2[C@]4(C)C[C@H]([C@@H](C2(C)C)O)O)(C)O)C)[C@@H]([C@H]([C@@H]1O)O)O	(Simões et al. 1999)
OCC1O[C@H](O[C@H]2[C@H](O)C[C@]3([C@H](C2(C)C)CC[C@@]2([C@@H]3CC=C3[C@@]2(C)CC[C@@]2([C@H]3[C@](C)(O)[C@H](C)CC2)C(=O)O)C)C)C([C@@H]([C@@H]1O)O)O	
CC(=C)[C@@H]1CC[C@]2([C@H]1[C@H]1CCC3[C@@]([C@]1(C)CC2)(C)CCC1[C@]3(C)CC[C@H]([C@@]1(C)C(=O)O)O)C(=O)O	(Li et al. 2007)
CO[C@@H]1CC[C@]2(C([C@]1(C)S(=O)(=O)O)CC[C@@]1(C2CC[C@H]2[C@@]1(C)CC[C@@]1([C@@H]2[C@@H](CC1)C(=C)C)C(=O)O)C)C	
CC(=O)O[C@H]1CC[C@]2(C(C1(C)C)CC[C@@]1(C2CC[C@H]2[C@@]1(C)CC[C@@]1([C@@H]2[C@@H](CC1)C(=C)C)C(=O)O)C)C	(Ryu et al. 1992)
COC(=O)[C@@]12CC[C@H]([C@@H]2[C@@H]2[C@](CC1)(C)[C@]1(C)CCC3[C@](C1CC2)(C)CC[C@@H](C3(C)C)O)C(=C)C	
COC(=O)[C@@]12CC[C@H]([C@@H]2[C@@H]2[C@](CC1)(C)[C@]1(C)CCC3[C@](C1CC2)(C)CC[C@@H](C3(C)C)OC(=O)C)C(=C)C	
C[C@@H]1CC[C@]2([C@H]([C@H]1C)C1=CCC3[C@@]([C@@]1(CC2)C)(C)CCC1[C@]3(C)C[C@@H](O)[C@H](C1(C)C)O)C(=O)O	
CC(=O)O[C@@H]1C[C@@]2(C)C(C([C@@H]1OC(=C)C)(C)C)CC[C@@]1(C2CC=C2[C@@]1(C)CC[C@@]1([C@@H]2[C@@H](C)[C@H](C)CC1)C(=O)O)C	
COC(=O)[C@@]12CC[C@H]([C@@H]([C@@H]2C2=CCC3[C@@]([C@@]2(CC1)C)(C)CCC1[C@]3(C)C[C@H]([C@H](C1(C)C)O)O)C)C	
COC(=O)[C@@]12CC[C@H]([C@@H]([C@@H]2C2=CCC3[C@@]([C@@]2(CC1)C)(C)CCC1[C@]3(C)C[C@H]([C@H](C1(C)C)OC(=O)C)OC(=O)C)C)C	
C[C@@H]1CC[C@]2([C@H]([C@H]1C)C1=CCC3[C@@]([C@@]1(CC2)C)(C)CCC1[C@]3(C)CC[C@@H](C1(C)C)O)C(=O)O	
CC(=O)O[C@H]1CC[C@]2(C(C1(C)C)CC[C@@]1(C2CC=C2[C@@]1(C)CC[C@@]1([C@@H]2[C@@H](C)[C@H](C)CC1)C(=O)O)C)C	
COC(=O)[C@@]12CC[C@H]([C@@H]([C@@H]2C2=CCC3[C@@]([C@@]2(CC1)C)(C)CCC1[C@]3(C)CC[C@@H](C1(C)C)O)C)C	
O=C1C=C2[C@@H]3[C@@H](C)[C@H](C)CC[C@@]3(CC[C@]2([C@]2([C@H]1[C@@]1(C)CC[C@@H](C([C@@H]1CC2)(C)C)O)C)C)C(=O)O	(Ryu et al. 1993)
C[C@@H]1CC[C@]2([C@H]([C@H]1C)C1=CC[C@H]3[C@@]([C@@]1(CC2)C)(C)CCC1[C@]3(C)C[C@@H](O)[C@H](C1(C)C)O)C(=O)O	
O[C@H]1COC([C@@H]([C@H]1O)O)O[C@@H]1CC[C@]2(C(C1(C)C)CC[C@@]1([C@@H]2CC=C2[C@@]1(C)CC[C@@]1([C@H]2[C@](C)(O)[C@H](C)CC1)C(=O)O)C)C	
OC[C@H]1O[C@@H](OC(=O)[C@@]23CC[C@H]([C@@]([C@H]3C3=CC[C@H]4[C@@]([C@@]3(CC2)C)(C)CCC2[C@]4(C)CC[C@H](C2(C)C)OC2OC[C@@H]([C@@H]([C@H]2O)O)O)(C)O)C)[C@@H]([C@H]([C@@H]1O)O)O	
C[C@@H]1CC[C@]2([C@@H]([C@]1(C)O)C1=CC[C@H]3[C@@]([C@@]1(CC2)C)(C)CC[C@@H]1[C@]3(C)CCC(=O)C1(C)C)C(=O)O	
O=C1C=C2[C@@H]3C[C@](C)(CC[C@]3(C)CC[C@]2([C@]2([C@H]1[C@@]1(C)CCC(=O)C([C@@H]1CC2)(C)C)C)C)C(=O)O	
C[C@@H]1CC[C@]2([C@@H]([C@H]1C)C1=CC[C@H]3[C@@]([C@@]1(CC2)C)(C)CC[C@@H]1[C@]3(C)CC[C@@H](C1(C)C)O)C(=O)O	
O[C@H]1CC[C@]2([C@H](C1(C)C)CC[C@@]1([C@@H]2CC=C2[C@@]1(C)CC[C@@]1([C@H]2[C@](C)(O)[C@H](C)CC1)C(=O)O)C)C	
O=C1C=C2[C@@H]3[C@@H](C)[C@H](C)CC[C@@]3(CC[C@]2([C@]2([C@H]1[C@@]1(C)CCC(=O)C([C@@H]1CC2)(C)C)C)C)C(=O)O	
N#CC[C@@]1(C)C(CC[C@@]2(C1CCC1[C@@]2(C)CC[C@@]2([C@@H]1[C@@H](CC2)C(=C)C)C(=O)O)C)C(C=O)(C)C	(Tolmacheva et al. 2019)
N#CC[C@@]1(C)C(CC[C@@]2(C1CCC1[C@@]2(C)CC[C@@]2([C@@H]1[C@@H](CC2)C(=C)C)C(=O)O)C)C(C(=O)O)(C)C	
CCC(NC(=O)[C@@]12CC[C@H]([C@@H]2C2[C@](CC1)(C)[C@]1(C)CCC([C@](C1CC2)(C)CC#N)C(C=O)(C)C)C(=C)C)CO	
CC[C@@H](NC(=O)[C@@]12CC[C@H]([C@@H]2C2[C@](CC1)(C)[C@]1(C)CCC([C@](C1CC2)(C)CC#N)C(C=O)(C)C)C(=C)C)CO	

CC[C@H](NC(=O)[C@@]12CC[C@H]([C@@H]2C2[C@](CC1)(C)[C@]1(C)CCC([C@](C1CC2)(C)CC#N)C(C=O)(C)C)C(=C)C)CO	
N#CC[C@@]1(C)C(CC[C@@]2(C1CCC1[C@@]2(C)CC[C@@]2([C@@H]1[C@@H](CC2)C(=C)C)C(=O)NC(CO)CC)C)C(C(=O)NC(CO)CC)(C)C	
N#CC[C@@]1(C)C(CC[C@@]2(C1CCC1[C@@]2(C)CC[C@@]2([C@@H]1[C@@H](CC2)C(=C)C)C(=O)OC)C)C(C(=O)NC(CO)CC)(C)C	
CCC(NC(=O)C(C1CC[C@@]2(C([C@@]1(C)CC#N)CCC1[C@@]2(C)CCC23[C@@H]1[C@@H](OC2)C(CC3)(C)C)C)(C)C)CO	
CC(=C)[C@@H]1CC[C@]2([C@H]1C1CCC3[C@@]([C@]1(C)CC2)(C)CCC1[C@]3(C)CCC(=O)C1(C)C)C(=O)O	
CCC(NC(=O)[C@@]12CC[C@H]([C@@H]2C2[C@](CC1)(C)[C@]1(C)CCC3[C@](C1CC2)(C)CCC(=O)C3(C)C)C(=C)C)CO	
CC[C@@H](NC(=O)[C@@]12CC[C@H]([C@@H]2C2[C@](CC1)(C)[C@]1(C)CCC3[C@](C1CC2)(C)CCC(=O)C3(C)C)C(=C)C)CO	
CC[C@H](NC(=O)[C@@]12CC[C@H]([C@@H]2C2[C@](CC1)(C)[C@]1(C)CCC3[C@](C1CC2)(C)CCC(=O)C3(C)C)C(=C)C)CO	
CCC(NC(=O)[C@@]12CC[C@H]([C@@H]2C2[C@](CC1)(C)[C@]1(C)CCC3[C@](C1CC2)(C)CC[C@@H](C3(C)C)O)C(=C)C)CO	
CC[C@@H](NC(=O)[C@@]12CC[C@H]([C@@H]2C2[C@](CC1)(C)[C@]1(C)CCC3[C@](C1CC2)(C)CC[C@@H](C3(C)C)O)C(=C)C)CO	
CC[C@H](NC(=O)[C@@]12CC[C@H]([C@@H]2C2[C@](CC1)(C)[C@]1(C)CCC3[C@](C1CC2)(C)CC[C@@H](C3(C)C)O)C(=C)C)CO	
CC[C@@H](NC(=O)[C@@]12CC[C@H]([C@@H]2C2[C@](CC1)(C)[C@]1(C)CCC3[C@](C1CC2)(C)CC[C@@H](C3(C)C)O)C(=C)C)COC(=O)C	
O/N=C\C(C1CC[C@@]2(C([C@@]1(C)CC#N)CCC1[C@@]2(C)CC[C@@]2([C@@H]1[C@@H](CC2)C(=C)C)C(=O)NC(C)C)C)(C)C	
CC[C@@H](NC(=O)[C@@]12CC[C@H]([C@@H]2C2[C@](CC1)(C)[C@]1(C)CCC3[C@](C1CC2)(C)CCC(=O)C3(C)C)C(=O)C)CO	
CC[C@H](NC(=O)[C@@]12CC[C@H]([C@@H]2C2[C@](CC1)(C)[C@]1(C)CCC3[C@](C1CC2)(C)CCC(=O)C3(C)C)C(=O)C)CO	
CC(CCC[C@H]([C@H]1CC[C@@H]2[C@]1(C)CC[C@H]1[C@H]2CC(=O)[C@@H]2[C@]1(C)C[C@H](O)[C@@H](C2)O)C)C	
[Na]OS(=O)(=O)O[C@H]1C[C@]2(C)[C@H]3CC[C@]4([C@H]([C@@H]3CC(=O)[C@H]2C[C@H]1OS(=O)(=O)O[Na])CC[C@@H]4[C@@H](CCCC(C)C)C)C	
CC(CCC[C@H]([C@H]1CC[C@@H]2[C@]1(C)CC[C@H]1[C@H]2CC(=O)[C@@H]2[C@]1(C)C[C@@H](O)[C@H](C2)O)C)C	
CC(CCC[C@H]([C@H]1CC[C@@H]2[C@]1(C)CC[C@H]1[C@H]2CC(=O)[C@@H]2[C@]1(C)C[C@@H](OS(=O)(=O)[O-])[C@H](C2)OS(=O)(=O)[O-])C)C.[Na+].[Na+]	
CC(CCC[C@H]([C@H]1CC[C@@H]2[C@]1(C)CC[C@H]1[C@H]2CC(=O)[C@@H]2[C@]1(C)C[C@H](O)[C@H](C2)O)C)C	
CC(CCC[C@H]([C@H]1CC[C@@H]2[C@]1(C)CC[C@H]1[C@H]2CC(=O)[C@@H]2[C@]1(C)C[C@H](OS(=O)(=O)[O-])[C@H](C2)OS(=O)(=O)[O-])C)C.[Na+].[Na+]	
O/N=C/1\C[C@H]2[C@@H]3CC[C@@H]([C@@]3(C)CC[C@@H]2[C@@]2([C@@H]1C[C@H](OS(=O)(=O)[O-])[C@H](C2)OS(=O)(=O)[O-])C)[C@@H](CCCC(C)C)C.[Na+].[Na+]	(Pujol et al. 2016)
CC(CCC[C@H]([C@H]1CC[C@@H]2[C@]1(C)CC[C@H]1[C@H]2C[C@H]([C@@H]2[C@]1(C)C[C@H](O)[C@H](C2)O)O)C)C	
CC(CCC[C@H]([C@H]1CC[C@@H]2[C@]1(C)CC[C@H]1[C@H]2C[C@H]([C@@H]2[C@]1(C)C[C@H](OS(=O)(=O)[O-])[C@H](C2)OS(=O)(=O)[O-])OS(=O)(=O)[O-])C)C.[Na+].[Na+].[Na+]	
CC(CCC[C@H]([C@H]1CC[C@@H]2[C@]1(C)CC[C@H]1[C@H]2C[C@@H]([C@@H]2[C@]1(C)C[C@H](O)[C@H](C2)O)O)C)C	
CC(CCC[C@H]([C@H]1CC[C@@H]2[C@]1(C)CC[C@H]1[C@H]2C[C@@H]([C@@H]2[C@]1(C)C[C@H](OS(=O)(=O)[O-])[C@H](C2)OS(=O)(=O)[O-])OS(=O)(=O)[O-])C)C.[Na+].[Na+].[Na+]	
CC(CCC[C@H]([C@H]1CC[C@@H]2[C@]1(C)CC[C@H]1[C@H]2C[C@H]([C@@H]2[C@]1(C)C[C@H](OS(=O)(=O)[O-])[C@H](C2)OS(=O)(=O)[O-])O)C)C.[Na+].[Na+]	
N#CC[C@@]1(C)C(CC[C@@]2(C1CC[C@H]1[C@@]2(C)CC[C@@]2([C@@H]1[C@@H](CC2)C(=C)C)C(=O)O)C)C(C=O)(C)C	
N#CC[C@@]1(C)C(CC[C@@]2(C1CC[C@H]1[C@@]2(C)CC[C@@]2([C@@H]1[C@@H](CC2)C(=C)C)C(=O)O)C)C(C(=O)O)(C)C	(Tolmacheva et al. 2014)
OCC(NC(=O)[C@@]12CC[C@H]([C@@H]2[C@@H]2[C@](CC1)(C)[C@]1(C)CC[C@H]([C@]([C@H]1CC2)(C)CC#N)C(C=O)(C)C)C(=C)C)CO	

OC[C@@H](CNC(=O)[C@@]12CC[C@H]([C@@H]2[C@@H]2[C@](CC1)(C)[C@]1(C)CC[C@H]([C@]([C@H]1CC2)(C)CC#N)C(C=O)(C)C)C(=C)C)O	
OC[C@H](C(C)C)NC(=O)[C@@]12CC[C@H]([C@@H]2[C@@H]2[C@](CC1)(C)[C@]1(C)CC[C@H]([C@]([C@H]1CC2)(C)CC#N)C(C=O)(C)C)C(=C)C	
N#CC[C@@]1(C)[C@@H](CC[C@@]2([C@@H]1CC[C@H]1[C@@]2(C)CC[C@@]2([C@@H]1[C@@H](CC2)C(=C)C)C(=O)NC(CO)(C)C)C)C(C=O)(C)C	
N#CC[C@@]1(C)[C@@H](CC[C@@]2([C@@H]1CC[C@H]1[C@@]2(C)CC[C@@]2([C@@H]1[C@@H](CC2)C(=C)C)C(=O)OC)C)C(C(=O)NC(CO)CO)(C)C	
OC[C@@H](CNC(=O)C([C@@H]1CC[C@@]2([C@@H]([C@@]1(C)CC#N)CC[C@H]1[C@@]2(C)CC[C@@]2([C@@H]1[C@@H](CC2)C(=C)C)C(=O)OC)C)(C)C)O	
N#CC[C@@]1(C)[C@@H](CC[C@@]2([C@@H]1CC[C@H]1[C@@]2(C)CC[C@@]2([C@@H]1[C@@H](CC2)C(=C)C)C(=O)OC)C)C(C(=O)N[C@H](C(C)C)CO)(C)C	
N#CC[C@@]1(C)[C@@H](CC[C@@]2([C@@H]1CC[C@H]1[C@@]2(C)CC[C@@]2([C@@H]1[C@@H](CC2)C(=C)C)C(=O)OC)C)C(C(=O)NC(CO)(C)C)(C)C	
OCC(NC(=O)C([C@@H]1CC[C@@]2([C@@H]([C@@]1(C)CC#N)CC[C@H]1[C@@]2(C)CC[C@@]23[C@H]1[C@@H](OC2)C(CC3)(C)C)C)(C)C)CO	
OCC(CNC(=O)C([C@@H]1CC[C@@]2([C@@H]([C@@]1(C)CC#N)CC[C@H]1[C@@]2(C)CC[C@@]23[C@H]1[C@@H](OC2)C(CC3)(C)C)C)(C)C)O	
OCC(C(C)C)CNC(=O)C([C@@H]1CC[C@@]2([C@@H]([C@@]1(C)CC#N)CC[C@H]1[C@@]2(C)CC[C@@]23[C@H]1[C@@H](OC2)C(CC3)(C)C)C)(C)C	
N#CC[C@@]1(C)[C@@H](CC[C@@]2([C@@H]1CC[C@H]1[C@@]2(C)CC[C@@]23[C@H]1[C@@H](OC2)C(C)(C)CC3)C)C(C(=O)NC(CO)(C)C)(C)C	
N#CC[C@@]1(C)[C@@H](CC[C@@]2([C@@H]1CC[C@H]1[C@@]2(C)CC[C@@]2([C@@H]1[C@@H](CC2)C(=C)C)C(=O)NC(CO)CO)C)C(C(=O)NC(CO)CO)(C)C	
N#CC[C@@]1(C)[C@@H](CC[C@@]2([C@@H]1CC[C@H]1[C@@]2(C)CC[C@@]2([C@@H]1[C@@H](CC2)C(=C)C)C(=O)NC[C@@H](CO)O)C)C(C(=O)N[C@@H](CO)O)(C)C	
N#CC[C@@]1(C)[C@@H](CC[C@@]2([C@@H]1CC[C@H]1[C@@]2(C)CC[C@@]2([C@@H]1[C@@H](CC2)C(=C)C)C(=O)NCC(C(C)C)CO)C)C(C(=O)N[C@H](C(C)C)CO)(C)C	
N#CCC1[C@@H](CC[C@@]2([C@@H]1CC[C@H]1[C@@]2(C)CC[C@@]2([C@@H]1[C@@H](CC2)C(=C)C)C(=O)OC)C)C(COC(=O)C)(C)C	
N#CC[C@@]1(C)[C@@H](CC[C@@]2([C@@H]1CC[C@H]1[C@@]2(C)CC[C@@]23[C@H]1[C@@H](OC2)C(C)(C)CC3)C)C(COC(=O)C)(C)C	
N#CCC1[C@@H](CC[C@@]2([C@@H]1CC[C@H]1[C@@]2(C)CC[C@@]2([C@@H]1[C@@H](CC2)C(=C)C)C(=O)OC)C)C(COC(=O)CCC(=O)O)(C)C	
N#CC[C@@]1(C)[C@@H](CC[C@@]2([C@@H]1CC[C@H]1[C@@]2(C)CC[C@@]23[C@H]1[C@@H](OC2)C(C)(C)CC3)C)C(COC(=O)CCC(=O)O)(C)C	
N#CCC1[C@@H](CC[C@@]2([C@@H]1CC[C@H]1[C@@]2(C)CC[C@@]2([C@@H]1[C@@H](CC2)C(=C)C)C(=O)OC)C)C(COC(=O)CC(C(=O)O)(C)C)(C)C	
N#CC[C@@]1(C)[C@@H](CC[C@@]2([C@@H]1CC[C@H]1[C@@]2(C)CC[C@@]23[C@H]1[C@@H](OC2)C(C)(C)CC3)C)C(COC(=O)CC(C(=O)O)(C)C)(C)C	
N#CCC1[C@@H](CC[C@@]2([C@@H]1CC[C@H]1[C@@]2(C)CC[C@@]2([C@@H]1[C@@H](CC2)C(=C)C)C(=O)OC)C)C(COC(=O)CCC(C(=O)O)(C)C)(C)C	
N#CC[C@@]1(C)[C@@H](CC[C@@]2([C@@H]1CC[C@H]1[C@@]2(C)CC[C@@]23[C@H]1[C@@H](OC2)C(C)(C)CC3)C)C(COC(=O)CCC(C(=O)O)(C)C)(C)C	
N#CCC1[C@@H](CC[C@@]2([C@@H]1CC[C@H]1[C@@]2(C)CC[C@@]2([C@@H]1[C@@H](CC2)C(=C)C)C(=O)OC)C)C(COC(=O)CC(CC(=O)O)(C)C)(C)C	
N#CC[C@@]1(C)[C@@H](CC[C@@]2([C@@H]1CC[C@H]1[C@@]2(C)CC[C@@]23[C@H]1[C@@H](OC2)C(C)(C)CC3)C)C(COC(=O)CCC(=O)OC)(C)C	
N#CCC1[C@@H](CC[C@@]2([C@@H]1CC[C@H]1[C@@]2(C)CC[C@@]2([C@@H]1[C@@H](CC2)C(=C)C)C(=O)OC)C)C(COC(=O)CC(C(=O)OC)(C)C)(C)C	
COC(=O)[C@@]12CC[C@H](C2C2[C@](CC1)(C)[C@]1(C)CCC3[C@](C1CC2)(C)C(=CC3(C)C)C(=O)O)C(=C)C	(Konysheva et al. 2017)
OC(=O)C1=CC(C2[C@@]1(C)C1CCC3[C@@]([C@@]1(CC2)C)(C)CC[C@@]12C3[C@@H](OC1)C(C)(C)CC2)(C)C	
COC(=O)[C@@]12CC[C@H](C2C2[C@](CC1)(C)[C@]1(C)CCC3[C@](C1CC2)(C)C(=C(C3(C)C)C)C(=O)O)C(=C)C	
OC(=O)C1=C(C)C(C2[C@@]1(C)C1CCC3[C@@]([C@@]1(CC2)C)(C)CC[C@@]12C3[C@@H](OC1)C(C)(C)CC2)(C)C	
NCC1=CC(C2[C@@]1(C)C1CCC3[C@@]([C@@]1(CC2)C)(C)CC[C@@]12C3[C@@H](OC1)C(C)(C)CC2)(C)C	
Oc1ccc(cc1)CC(C(=O)O)NC(=O)C1O[C@H](O[C@H]2CC[C@]3([C@H](C2(C)C)CC[C@@]2([C@@H]3C(=O)C=C3[C@@]2(C)CC[C@@]2([C@H]3C[C@](C)(CC2)C(=O)NC(C(=O)O)Cc2ccc(cc2)O)C)C)C)C([C@@H]([C@@H]1O)O)O[C@@H]1OC(C(=O)NC(C(=O)O)Cc2ccc(cc2)O)[C@H]([C@H](C1O)O)O	(Baltina, L. A., Jr. et al. 2017)

O[C@H]1CC[C@]2([C@H](C1(C)C)CC[C@@]1([C@@H]2CC=C2[C@@]1(C)CC[C@@]1([C@H]2CC(C)(C)CC1)C)C)C	(Kuo et al. 2011)
C=C(C(C)C)CC[C@H]([C@H]1CC[C@@]2([C@]1(C)CC[C@@]13[C@H]2CC[C@@H]2[C@]3(C1)CC[C@@H]([C@H]2C)O)C)C	
OC[C@@]12[C@H](O)C[C@@H](C[C@@]2(O)CC[C@@H]2[C@@H]1[C@H](O)C[C@]1([C@]2(O)CC[C@@H]1C1=CC(=O)OC1)C)O[C@@H]1O[C@@H](C)[C@@H]([C@H]([C@H]1O)O)O.O.O.O.O.O.O.O.O.O	(Su et al. 2008)
OC[C@@]12[C@H](O)C[C@@H](C[C@@]2(O)CC[C@@H]2[C@@H]1[C@H](O)C[C@]1([C@]2(O)CC[C@@H]1C1=CC(=O)OC1)C)O[C@@H]1O[C@@H](C)[C@@H]([C@H]([C@H]1O)O)O	
O[C@H]1CC[C@]2([C@@H](C1)CC[C@@H]1[C@@H]2CC[C@]2([C@]1(O)C[C@@H]([C@@H]2C1=CC(=O)OC1)O)C)C	
OC[C@@]12CC[C@@H](C[C@@]2(O)CC[C@@H]2[C@@H]1CC[C@]1([C@]2(O)CC[C@@H]1C1=CC(=O)OC1)C)O	
O=C1OCC(=C1)[C@H]1CC[C@]2([C@]1(C)CC[C@H]1[C@H]2CC[C@H]2[C@]1(C)CC[C@@H](C2)O[C@H]1C[C@H](O)[C@@H]([C@H](O1)C)O)O	(Bertol et al. 2011)
O=C1OCC(=C1)[C@H]1CC[C@]2([C@]1(C)CCC1C2CC[C@H]2[C@]1(C)CC[C@@H](C2)O[C@H]1C[C@H](O)[C@@H]([C@H](O1)C)O[C@H]1C[C@H](O)[C@@H]([C@H](O1)C)O)O	
OC[C@H]1O[C@@H](O[C@H]2[C@@H](O)C[C@@H](O[C@@H]2C)O[C@H]2CC[C@]3([C@@H](C2)CC[C@@H]2[C@@H]3CC[C@]3([C@]2(O)CC[C@@H]3C2=CC(=O)OC2)C)C)[C@@H]([C@H]([C@@H]1O)O)O	
CC(=O)OC1C(O)CC(OC1C)OC1C(O)CC(OC1C)OC1C(O)CC(OC1C)OC1CCC2(C(C1)CCC1C2CCC2(C1(O)CCC2C1=CC(=O)OC1)C)C	
OC[C@H]1O[C@@H](O[C@@H]2[C@@H](C)O[C@H](C[C@@H]2OC(=O)C)O[C@H]2[C@@H](O)C[C@@H](O[C@@H]2C)O[C@H]2[C@@H](O)C[C@@H](O[C@@H]2C)O[C@H]2CC[C@]3([C@@H](C2)CC[C@@H]2[C@@H]3CC[C@]3([C@]2(O)CC[C@@H]3C2=CC(=O)OC2)C)C)[C@@H]([C@H]([C@@H]1O)O)O	
OC[C@H]1O[C@@H](O[C@H]2[C@@H](O)C[C@@H](O[C@@H]2C)O[C@H]2CC[C@]3([C@@H](C2)CC[C@@H]2[C@@H]3CC[C@]3([C@]2(O)C[C@@H]([C@@H]3C2=CC(=O)OC2)O)C)C)[C@@H]([C@H]([C@@H]1O)O)O	
O=C1OCC(=C1)[C@H]1CC[C@]2([C@]1(C)[C@H](O)C[C@H]1[C@H]2CC[C@H]2[C@]1(C)CC[C@@H](C2)O[C@H]1C[C@H](O)[C@@H]([C@H](O1)C)O[C@H]1C[C@H](O)[C@@H]([C@H](O1)C)O)O	
OC[C@H]1C[C@@H](O[C@H]2[C@@H](O)C[C@@H](O[C@@H]2C)O[C@H]2[C@@H](O)C[C@@H](O[C@@H]2C)O[C@H]2[C@@H](O)C[C@@H](O[C@@H]2C)O[C@H]2CC[C@]3([C@@H](C2)CC[C@@H]2[C@@H]3C[C@@H](O)[C@]3([C@]2(O)CC[C@@H]3C2=CC(=O)OC2)C)C)[C@@H]([C@H]([C@@H]1O)O)O	
CO[C@H]1C[C@H](O[C@H]2CC[C@]3([C@](C2)(O)CC[C@@H]2[C@@H]3CC[C@]3([C@]2(O)CCC3C2=CC(=O)OC2)C)C=O)O[C@@H]([C@H]1O[C@@H]1O[C@H](CO[C@@H]2O[C@H](CO)[C@H]([C@@H]([C@H]2O)O)O)[C@H]([C@@H]([C@H]1O)O)O)C	
CO[C@H]1C[C@H](O[C@H]2[C@@H](O)C[C@@H](O[C@@H]2C)O[C@H]2[C@@H](O)C[C@@H](O[C@@H]2C)O[C@H]2CC[C@]3([C@@H](C2)CC[C@@H]2[C@@H]3CC[C@]3([C@]2(O)CC[C@@H]3C2=CC(=O)OC2)C)C)O[C@@H]([C@H]1O)C	
CO[C@H]1[C@@H](O)C[C@@H](O[C@@H]1C)O[C@H]1[C@@H](O)C[C@@H](O[C@@H]1C)O[C@H]1[C@@H](O)C[C@@H](O[C@@H]1C)O[C@H]1CC[C@]2([C@@H](C1)CC[C@@H]1[C@@H]2CC[C@]2([C@]1(O)CC[C@@H]2C1=CC(=O)OC1)C)C	
O=C[C@@]12CC[C@@H](C[C@@]2(O)CC[C@@H]2[C@@H]1CC[C@]1([C@]2(O)CC[C@@H]1C1=CC(=O)OC1)C)O[C@@H]1O[C@@H](O)[C@@H]([C@H]([C@H]1O)O)O	
O=C[C@@]12CC[C@@H](C[C@@]2(O)CC[C@@H]2[C@@H]1CC[C@]1([C@]2(O)CC[C@@H]1C1=CC(=O)OC1)C)O[C@H]1C[C@H](O)[C@@H]([C@H](O1)C)O	
CO[C@H]1C[C@H](O[C@H]2CC[C@]3([C@](C2)(O)CC[C@@H]2[C@@H]3CC[C@]3([C@]2(O)CC[C@@H]3C2=CC(=O)OC2)C)C=O)O[C@@H]([C@H]1O)C	
O[C@H]1CC[C@]2([C@@H](C1)CC[C@@H]1[C@@H]2C[C@@H](O)[C@]2([C@]1(O)CC[C@@H]2C1=CC(=O)OC1)C)C	
O=C1OCC(=C1)[C@H]1CC[C@]2([C@]1(C)[C@H](O)C[C@H]1[C@H]2CC[C@H]2[C@]1(C)CC[C@@H](C2)O[C@H]1C[C@H](O)[C@@H]([C@H](O1)C)O[C@H]1C[C@H](O)[C@@H]([C@H](O1)C)O[C@H]1C[C@H](O)[C@@H]([C@H](O1)C)O)O	
O=C1OCC(=C1)[C@H]1CC[C@]2([C@]1(C)CC[C@H]1[C@H]2CC[C@H]2[C@]1(C)CC[C@@H](C2)O[C@H]1C[C@H](O)[C@@H]([C@H](O1)C)O[C@H]1C[C@H](O)[C@@H]([C@H](O1)C)O[C@H]1C[C@H](O)[C@@H]([C@H](O1)C)O)O	
O[C@H]1CC[C@]2(C(=CC[C@@H]3[C@@H]2CC[C@]2([C@H]3CC[C@@H]2C(=O)C)C)C1)C	(Davola et al. 2015)
CC(=O)O[C@H]1CC[C@]2(C(=CCC3C2CC[C@]2(C3C[C@H]([C@@H]2C(=O)C)C(=O)O)C)C1)C	

CC(=O)O[C@H]1CC[C@]2(C(=CCC3C2CC[C@]2(C3C[C@H]([C@@H]2C(=O)C)C(=O)Nc2ccccc2)C)C1)C	
CC(=O)O[C@H]1CC[C@]2(C(=CCC3C2CC[C@]2(C3C[C@H]([C@@H]2C(=O)C)C(=O)N(Cc2ccccc2)CC(=O)NC(C)(C)C)C)C1)C	
CC(=O)O[C@H]1CC[C@]2(C(=CCC3C2CC[C@]2(C3C[C@H]([C@@H]2C(=O)C)C(=O)N(c2ccccc2)CC(=O)NC(C)(C)C)C)C1)C	
CC(=O)O[C@H]1CC[C@]2(C(=CCC3C2CC[C@]2(C3C[C@H]([C@@H]2C(=O)C)C(=O)N(c2ccc(cc2)Cl)CC(=O)NC(C)(C)C)C)C1)C	
CC(=O)O[C@H]1CC[C@]2(C(=CCC3C2CC[C@]2(C3C[C@H]([C@@H]2C(=O)C)C(=O)N(c2ccc(cc2)F)CC(=O)NC(C)(C)C)C)C1)C	
O=C(NC(C)(C)C)CN(C(=O)[C@@H]1CC2[C@]([C@H]1C(=O)C)(C)CCC1C2CC=C2[C@]1(C)CC[C@@H](C2)O)Cc1ccccc1	
O=C(NC(C)(C)C)CN(C(=O)[C@@H]1CC2[C@]([C@H]1C(=O)C)(C)CCC1C2CC=C2[C@]1(C)CC[C@@H](C2)O)c1ccc(cc1)Cl	
O=C(NC(C)(C)C)CN(C(=O)[C@@H]1CC2[C@]([C@H]1C(=O)C)(C)CCC1C2CC=C2[C@]1(C)CC[C@@H](C2)O)c1ccc(cc1)F	
CC(=O)O[C@H]1CC[C@]2([C@H](C1(C)C)CC[C@@]1([C@@H]2C(=O)C=C2[C@@]1(C)CC[C@@]1([C@H]2C[C@](C)(CC1)C(=O)O)C)C)C	
OCCNC(=O)[C@@]1(C)CC[C@]2([C@@H](C1)C1=CC(=O)[C@H]3[C@@]([C@@]1(CC2)C)(C)CC[C@@H]1[C@]3(C)CC[C@@H](C1(C)C)OC(=O)C)C	
OCCCCNC(=O)[C@@]1(C)CC[C@]2([C@@H](C1)C1=CC(=O)[C@H]3[C@@]([C@@]1(CC2)C)(C)CC[C@@H]1[C@]3(C)CC[C@@H](C1(C)C)OC(=O)C)C	(Zígolo et al. 2018)
OCC(NC(=O)[C@@]1(C)CC[C@]2([C@@H](C1)C1=CC(=O)[C@H]3[C@@]([C@@]1(CC2)C)(C)CC[C@@H]1[C@]3(C)CC[C@@H](C1(C)C)OC(=O)C)C)C	
CC(CNC(=O)[C@@]1(C)CC[C@]2([C@@H](C1)C1=CC(=O)[C@H]3[C@@]([C@@]1(CC2)C)(C)CC[C@@H]1[C@]3(C)CC[C@@H](C1(C)C)OC(=O)C)C)O	
CC(=C)[C@@H]1CC[C@]2([C@H]1[C@H]1CC[C@H]3[C@@]([C@]1(C)CC2)(C)CC[C@@H]1[C@]3(C)CC[C@H]([C@@]1(C)C(=O)O)O)C(=O)O	
CC(=C)[C@@H]1CC[C@]2([C@H]1[C@H]1CC[C@H]3[C@@]([C@]1(C)CC2)(C)CC[C@@H]1[C@]3(C)CC[C@H](C1(C)C)OS(=O)(=O)O)C(=O)O	
CC(=C)[C@@H]1CC[C@]2([C@H]1[C@H]1CC[C@H]3[C@@]([C@]1(C)CC2)(C)CC[C@@H]1[C@]3(C)CC[C@@H](C1(C)C)O)C(=O)O	(Visalli et al. 2015)
CC(=C)[C@@H]1CC[C@]2([C@H]1[C@H]1CC[C@H]3[C@@]([C@]1(C)CC2)(C)CC[C@@H]1[C@]3(C)CCC(=O)C1(C)C)C(=O)O	
OC[C@@]12CC[C@H]([C@@H]2[C@@H]2[C@](CC1)(C)[C@]1(C)CC[C@@H]3[C@]([C@H]1CC2)(C)CC[C@@H](C3(C)C)O)C(=C)C	
CC(=O)OCC1O[C@@H](Oc2nnn(c2)[C@H]2CC[C@]3([C@@H](C2)CC[C@@H]2[C@@H]3CC[C@]3([C@]2(O)CC[C@@H]3C2=CC(=O)OC2)C)C)C([C@@H]([C@@H]1OC(=O)C)OC(=O)C)OC(=O)C	
CC(=O)OCC1O[C@H](Oc2nnn(c2)[C@H]2CC[C@]3([C@@H](C2)CC[C@@H]2[C@@H]3CC[C@]3([C@]2(O)CC[C@@H]3C2=CC(=O)OC2)C)C)C([C@@H]([C@@H]1OC(=O)C)OC(=O)C)(OC(=O)C)OC(=O)C	
CC(=O)OCC1O[C@@H](Oc2nnn(c2)[C@H]2CC[C@]3([C@@H](C2)CC[C@@H]2[C@@H]3CC[C@]3([C@]2(O)CC[C@@H]3C2=CC(=O)OC2)C)C)C([C@@H]([C@@H]1O[C@@H]1OC(COC(=O)C)[C@H]([C@H](C1OC(=O)C)OC(=O)C)OC(=O)C)OC(=O)C)OC(=O)C	
OCC1O[C@@H](Oc2nnn(c2)[C@H]2CC[C@]3([C@@H](C2)CC[C@@H]2[C@@H]3CC[C@]3([C@]2(O)CC[C@@H]3C2=CC(=O)OC2)C)C)C([C@@H]([C@@H]1O)O)O	
OCC1O[C@@H](Oc2nnn(c2)[C@H]2CC[C@]3([C@@H](C2)CC[C@@H]2[C@@H]3CC[C@]3([C@]2(O)CC[C@@H]3C2=CC(=O)OC2)C)C)C([C@@H]([C@H]1O)O)O	
OCC1O[C@H](Oc2nnn(c2)[C@H]2CC[C@]3([C@@H](C2)CC[C@@H]2[C@@H]3CC[C@]3([C@]2(O)CC[C@@H]3C2=CC(=O)OC2)C)C)C([C@@H]([C@@H]1O)O)(O)O	(Boff et al. 2019)
OCC1O[C@@H](Oc2nnn(c2)[C@H]2CC[C@]3([C@@H](C2)CC[C@@H]2[C@@H]3CC[C@]3([C@]2(O)CC[C@@H]3C2=CC(=O)OC2)C)C)C([C@@H]([C@@H]1O[C@@H]1OC(CO)[C@H]([C@H](C1O)O)O)O)O	
ClCC(=O)N[C@H]1CC[C@]2([C@@H](C1)CC[C@@H]1[C@@H]2CC[C@]2([C@]1(O)CC[C@@H]2C1=CC(=O)OC1)C)C	
OCCNCC(=O)N[C@H]1CC[C@]2([C@@H](C1)CC[C@@H]1[C@@H]2CC[C@]2([C@]1(O)CC[C@@H]2C1=CC(=O)OC1)C)C	
OCC(=O)N[C@H]1CC[C@]2([C@@H](C1)CC[C@@H]1[C@@H]2CC[C@]2([C@]1(O)CC[C@@H]2C1=CC(=O)OC1)C)C	
N#Cc1ccc(cc1)NCC(=O)N[C@H]1CC[C@]2([C@@H](C1)CC[C@@H]1[C@@H]2CC[C@]2([C@]1(O)CC[C@@H]2C1=CC(=O)OC1)C)C	
O=C(N[C@H]1CC[C@]2([C@@H](C1)CC[C@@H]1[C@@H]2CC[C@]2([C@]1(O)CC[C@@H]2C1=CC(=O)OC1)C)C)CN1CCC(CC1)c1ccccc1	

O=C(N[C@H]1CC[C@]2([C@@H](C1)CC[C@@H]1[C@@H]2CC[C@]2([C@]1(O)CC[C@@H]2C1=CC(=O)OC1)C)C)CNc1ccc(cc1)Cl	
O=C1OCC(=C1)[C@H]1CC[C@]2([C@]1(C)CC[C@H]1[C@H]2CC[C@H]2[C@]1(C)CC[C@@H](C2)NCCC(C(C(O)C)O)O)O	
O[C@H]1CC[C@]2([C@@H](C1)CC[C@@H]1[C@@H]2CC[C@]2([C@]1(O)CC[C@@H]2C1=CC(=O)OC1)C)C	
CC(=C)[C@@H]1CC[C@]2([C@H]1[C@H]1CC[C@H]3[C@@]([C@]1(C)CC2)(C)CC[C@@H]1[C@]3(C)CC[C@@H](C1(C)C)O)C	(Heidary Navid et al. 2014)
C[C@@H]1CC[C@]2([C@@H]([C@H]1C)C1=CC[C@H]3[C@@]([C@@]1(CC2)C)(C)CC[C@@H]1[C@]3(C)CC[C@H]([C@]1(C)C(=O)O)O)C	(Goswami et al. 2018)
C/C=C/1\C(=O)C[C@@H]2[C@]1(C)CC[C@H]1[C@H]2CC[C@@H]2[C@]1(C)C[C@H](O)[C@@H](C2)O	
O[C@@H]([C@@H](C(O)(C)C)O)C[C@H]([C@H]1CC[C@@]2([C@]1(C)CC[C@@]13[C@H]2CC[C@@H]2[C@]3(C1)CCC(=O)C2(C)C)C)C	(Joycharat et al. 2008)
C/C=C\1/C(=O)C[C@@H]2[C@]1(C)CC[C@H]1[C@H]2CC[C@@H]2[C@]1(C)C[C@H](O)[C@@H](C2)O	
COC(=O)CC[C@@]12C[C@@]32CC[C@]2([C@@]([C@@H]3CC[C@H]1C(O)(C)C)(C)C[C@H]1[C@@H]2[C@H](C)C[C@@]2(O1)OC(=O)C(=C2)C)C	
CC1=C[C@@]2(OC1=O)O[C@H]1C[C@@]3([C@]([C@H]1[C@@H](C2)C)(C)CC[C@@]12[C@H]3CC[C@@H]3[C@]2(C1)CCC(=O)C3(C)C)C	
COC(=O)CC[C@@]12C[C@@]32CC[C@]2([C@@]([C@@H]3CC[C@H]1C(O)(C)C)(C)C[C@@H]1[C@@H]2[C@H](C)C[C@]2(O1)OC(=O)[C@H](C2)C)C	
O=C(C[C@@H](C(=O)O)C)C[C@H]([C@H]1CC[C@@]2([C@]1(C)CC[C@@]13[C@H]2CC[C@@H]2[C@]3(C1)CCC(=O)C2(C)C)C)C	
COC(=O)CC[C@@]12C[C@@]32CC[C@]2([C@@]([C@@H]3CC[C@H]1C(O)(C)C)(C)CC[C@@H]2[C@@H](CC(=O)C[C@@H](C(=O)O)C)C)C	
COC(=O)CC[C@@]12C[C@@]32CC[C@]2([C@@]([C@@H]3CC[C@H]1C(O)(C)C)(C)C[C@@H]1[C@@H]2[C@H](C)C[C@]2(O1)OC(=O)[C@@H](C2)C)C	(Zhao et al. 2020)
OC(=O)CC[C@@]12C[C@@]32CC[C@]2([C@@]([C@@H]3CC[C@H]1C(O)(C)C)(C)C[C@@H]1[C@@H]2[C@H](C)C[C@]2(O1)OC(=O)[C@@H](C2)C)C	
O=C(/C=C(/C(=O)O)\C)C[C@H]([C@H]1CC[C@@]2([C@]1(C)CC[C@@]13[C@H]2CC[C@@H]2[C@]3(C1)CCC(=O)C2(C)C)C)C	
O=C1C=C2O[C@@]3(C=C2[C@H](C(O1)(C)C)CCC[C@]1([C@@](CCC3)(C)[C@@H]2[C@@H](C1)O[C@]1(C[C@H]2C)C[C@H](C(=O)O1)C)C)O	
O=C1C=C2O[C@@H]3C=C2[C@H](C(O1)(C)C)CCC[C@]1([C@@](CCC3)(C)[C@@H]2[C@@H](C1)O[C@]1(C[C@H]2C)C[C@H](C(=O)O1)C)C	
CC[C@H]([C@H]([C@@H]([C@H](C1CC[C@@H]2[C@]1(C)CC[C@H]1[C@H]2CC(=O)[C@@]2([C@]1(C)CC[C@@H](C2)Br)O)C)O)O)C(C)C	
CC[C@H]([C@H]([C@@H]([C@H](C1CC[C@@H]2[C@]1(C)CC[C@H]1[C@H]2CC(=O)[C@@]2([C@]1(C)CC[C@@H](C2)O)F)C)O)O)C(C)C	(Michelini et al. 2004)
CC[C@H]([C@H]([C@@H]([C@H](C1CC[C@@H]2[C@]1(C)CC[C@H]1[C@H]2CC(=O)[C@@]2([C@]1(C)CC[C@@H](C2)O)O)C)O)O)C(C)C	
OC[C@H]1O[C@@H](OC[C@H]2O[C@@H](OC[C@H]3O[C@@H](O[C@H]4[C@@H](O)C[C@]5([C@H](C4(CO)CO)CC[C@@]4([C@@H]5CC=C5[C@@]4(C)C[C@H]([C@@]4([C@H]5CC(C)(C)CC4)C(=O)O[C@@H]4OC[C@@H]([C@@H]([C@H]4O[C@@H]4O[C@@H](C)[C@@H]([C@H]([C@H]4O)O)O[C@@H]4OC[C@H]([C@@H]([C@H]4O)O)O)O)O)O)C)C)[C@@H]([C@H]([C@@H]3O)O)O)[C@@H]([C@H]([C@@H]2O)O)O)[C@@H]([C@H]([C@@H]1O)O)O	
OC[C@H]1O[C@@H](OC[C@H]2O[C@@H](OC[C@H]3O[C@@H](O[C@H]4[C@@H](O)C[C@]5([C@H](C4(CO)CO)CC[C@@]4([C@@H]5CC=C5[C@@]4(C)C[C@H]([C@@]4([C@H]5CC(C)(C)CC4)C(=O)O[C@@H]4OC[C@@H]([C@@H]([C@H]4O[C@@H]4O[C@@H](C)[C@@H]([C@H]([C@H]4O)O)O)O)O)C)C)[C@@H]([C@H]([C@@H]3O)O)O)[C@@H]([C@H]([C@@H]2O)O)O)[C@@H]([C@H]([C@@H]1O)O)O	
OC[C@H]1O[C@@H](OC[C@H]2O[C@@H](O[C@H]3[C@@H](O)C[C@]4([C@H]([C@]3(C)CO)CC[C@@]3([C@@H]4CC=C4[C@@]3(C)C[C@H]([C@@]3([C@H]4CC(C)(C)CC3)C(=O)O[C@@H]3OC[C@@H]([C@@H]([C@H]3O[C@@H]3O[C@@H](C)[C@@H]([C@H]([C@H]3O)O)O[C@@H]3OC[C@H]([C@@H]([C@H]3O)O[C@@H]3OC[C@]([C@H]3O)(O)CO)O)O)O)O)C)C)[C@@H]([C@H]([C@@H]2O)O)O)[C@@H]([C@H]([C@@H]1O)O)O	(He et al. 2005)
OC[C@H]1O[C@@H](OC[C@H]2O[C@@H](O[C@H]3[C@@H](O)C[C@]4([C@H](C3(CO)CO)CC[C@@]3([C@@H]4CC=C4[C@@]3(C)C[C@H]([C@@]3([C@H]4CC(C)(C)CC3)C(=O)O[C@@H]3OC[C@@H]([C@@H]([C@H]3O[C@@H]3O[C@@H](C)[C@@H]([C@H]([C@H]3O)O)O[C@@H]3OC[C@H]([C@@H]([C@H]3O)O[C@@H]3OC[C@]([C@H]3O)(O)CO)O)O)O)O)C)C)[C@@H]([C@H]([C@@H]2O)O)O)[C@@H]([C@H]([C@@H]1O)O)O	

OC[C@H]1O[C@@H](OC[C@H]2O[C@@H](OC[C@H]3O[C@@H](O[C@H]4[C@@H](O)C[C@]5([C@H](C4(CO)CO)CC[C@@]4([C@@H]5CC=C5[C@@]4(C)C[C@H]([C@@]4([C@H]5CC(C)(C)CC4)C(=O)O[C@@H]4OC[C@@H]([C@@H]([C@H]4O[C@@H]4O[C@@H](C)[C@@H]([C@H]([C@H]4O)O)O[C@@H]4OC[C@H]([C@@H]([C@H]4O)O[C@@H]4OC[C@]([C@H]4O)(O)CO)O)O)O)O)C)C)[C@@H]([C@H]([C@@H]3O)O)O)[C@@H]([C@H]([C@@H]2O)O)O)[C@@H]([C@H]([C@@H]1O)O)O	
N#CC[C@@H](C(C1CCC2C(C1=C)CCC1[C@@]2(C)CC[C@@]23C1[C@@H](OC2)C(CC3)(C)C)(C)C)O	(Tolmacheva et al. 2017)
N#C[C@H]([C@@H](C(C1CCC2C(C1=C)CCC1[C@@]2(C)CC[C@@]23C1[C@@H](OC2)C(CC3)(C)C)(C)C)Cl)O	
COC(=O)[C@]1(C)CC[C@]2([C@@H](C1)[C@]1(C)CC[C@@]3(C(=CC(=C4C3=CC(=O)C(=C4)O)O)[C@]1(CC2)C)C)C	(Liu et al. 2019)
COC(=O)[C@]1(C)CC[C@]2([C@@H](C1)[C@]1(C)CC[C@@]3(C(=CC(=C4C3=CC(=O)C(=C4C)O)O)[C@]1(CC2)C)C)C	
COC(=O)C([C@@H]1CC[C@@]2([C@@H]([C@@]1(C)CC(=O)O)CC=C1[C@@]2(C)CC[C@@]2([C@H]1[C@](C)(O)[C@@H](CC2)CO)C(=O)O)C)(C)C	
OCC1=C(C)[C@@H]2[C@](CC1)(CC[C@@]1(C2=CC[C@H]2[C@@]1(C)CC[C@@H]1[C@]2(C)CCC(=O)[C@]1(C)CO)C)C(=O)O	
OC[C@@]1(C)[C@H](O)[C@H](O)C[C@]2([C@H]1CC[C@@]1([C@@H]2CC=C2[C@@]1(C)CC[C@@]1(C2=C(C)[C@@H](CC1)C)C(=O)O)C)C	
OC[C@@H]1CC[C@]2([C@@H]([C@H]1C)C1=CC[C@H]3[C@@]([C@@]1(CC2)C)(C)[C@@H](O)C[C@@H]1[C@]3(C)CCC(=O)C1(C)C)C(=O)O	
COc1cc(/C=C/C(=O)O[C@H]2CC[C@]3([C@H](C2(C)C)CC[C@@]2([C@@H]3CC=C3[C@@]2(C)CC[C@@]2([C@H]3[C@@H](O)[C@H](C)CC2)C(=O)O)C)C)ccc1O	
CC(=O)[C@@H]1CC[C@]2([C@H]1[C@H]1CC[C@H]3[C@@]([C@]1(C)CC2)(C)[C@@H](O)C[C@@H]1[C@]3(C)CCC(=O)C1(C)C)C(=O)O	
CC(=O)[C@@H]1CC[C@]2([C@H]1[C@H]1CC[C@H]3[C@@]([C@]1(C)CC2)(C)C[C@@H]([C@@H]1[C@]3(C)CCC(=O)C1(C)C)O)C(=O)O	
COc1cc(/C=C/C(=O)O[C@H]2CC[C@]3([C@H](C2(C)C)CC[C@@]2([C@@H]3CC[C@H]3[C@@]2(C)CC[C@@]2([C@@H]3[C@@H](CC2)C(O)(C)C)C(=O)O)C)C)ccc1O	
COc1cc(/C=C/C(=O)O[C@H]2[C@H](O)C[C@]3([C@H](C2(C)C)CC[C@@]2([C@@H]3CC=C3[C@@]2(C)CC[C@@]2([C@H]3C[C@H](C)CC2)C(=O)O)C)C)ccc1O	
OC[C@]1(C)[C@@H](OC(=O)/C=C/c2ccc(c(c2)OC)O)[C@H](O)C[C@]2([C@H]1CC[C@@]1([C@@H]2CC=C2[C@@]1(C)CC[C@@]1([C@H]2CC(CC1)(C)C)C(=O)O)C)C	
COc1cc(/C=C/C(=O)O[C@H]2[C@H](O)C[C@]3([C@H](C2(C)C)CC[C@@]2([C@@H]3CC[C@H]3[C@@]2(C)CC[C@@]2([C@@H]3[C@@H](CC2)C(=C)C)C(=O)O)C)C)ccc1O	
OC[C@]1(C)[C@@H](O)CC[C@]2([C@H]1CC[C@@]1(C2=CC(=O)[C@H]2[C@@]1(C)CC[C@@]1([C@H]2[C@@H](C)[C@@H](CC1)C)C)C)C	
OC[C@@]1(C)[C@@H](O)CC[C@]2([C@H]1CC[C@@]1(C2=CC(=O)[C@H]2[C@@]1(C)CC[C@@]1([C@H]2[C@@H](C)[C@@H](CC1)C)C)C)C	
C[C@@H]1CC[C@]2([C@@H]([C@H]1C)[C@H]1C(=O)C=C3[C@@]([C@@]1(CC2)C)(C)CC[C@@H]1[C@]3(C)CC[C@@H]([C@]1(C)C(=O)O)O)C	
CCOCC1=C(C)[C@@H]2[C@](CC1)(CC[C@@]1(C2=CC[C@H]2[C@@]1(C)CC[C@@H]1[C@]2(C)C[C@@H](O)[C@H]([C@@]1(C)CO)O)C)C(=O)O	
CCOCC1=C(C)[C@@H]2[C@](CC1)(CC[C@@]1(C2=CC[C@H]2[C@@]1(C)CC[C@@H]1[C@]2(C)CC[C@H]([C@]1(C)CO)O)C)C(=O)O	
OC[C@@]1(C)C(=O)CC[C@]2([C@H]1CC[C@@]1([C@@H]2CC=C2[C@@]1(C)CC[C@@]1([C@H]2[C@](C)(O)[C@@H](C[C@@H]1O)C)C(=O)O)C)C	
OC[C@@]1(C)[C@@H](O)CC[C@]2([C@H]1CC[C@@]1([C@@H]2C=CC2=C[C@@](CC[C@@]12C)(CCC(C(=O)C)C)C(=O)O)C)C	
COc1cc(/C=C\C(=O)O[C@H]2CC[C@]3([C@H](C2(C)C)CC[C@@]2([C@@H]3CC=C3[C@@]2(C)CC[C@@]2([C@H]3[C@@H](O)[C@H](C)CC2)C(=O)O)C)C)ccc1O	
COc1cc(/C=C\C(=O)O[C@H]2[C@H](O)C[C@]3([C@H](C2(C)C)CC[C@@]2([C@@H]3CC=C3[C@@]2(C)CC[C@@]2([C@H]3CC(C)(C)CC2)C(=O)O)C)C)ccc1O	
OC[C@]1(C)[C@H](O)[C@H](O)C[C@]2([C@H]1CC[C@@]1([C@@H]2[C@@H]2O[C@@H]2[C@]23[C@@]1(C)CC[C@@]1([C@H]3CC(CC1)(C)C)C(=O)O2)C)C	
COc1cc(/C=C\C(=O)O[C@H]2[C@H](O)C[C@]3([C@H](C2(C)C)CC[C@@]2([C@@H]3CC[C@H]3[C@@]2(C)CC[C@@]2([C@@H]3[C@@H](CC2)C(=C)C)C(=O)O)C)C)ccc1O	
OC[C@]1(C)[C@H](O)[C@H](O)C[C@]2([C@H]1CC[C@@]1([C@@H]2CC=C2[C@@]1(C)CC[C@@]1(C2=C(C)[C@@H](CC1)C)C(=O)O)C)C	
CC(=O)C(CC[C@@]1(CC[C@@]2(C(=C1)C=C[C@H]1[C@@]2(C)CC[C@@H]2[C@]1(C)C[C@H]([C@@H](C2(C)C)O)O)C)C(=O)O)C	
C[C@@H]1CC[C@]2([C@@H]([C@H]1C)C1=CC[C@H]3[C@@]([C@@]1(CC2)C)(C)CC[C@@H]1[C@]3(C)C[C@@H](O)[C@@H](C1(C)C)O)C(=O)O	

OC[C@]1(C)C(=O)CC[C@]2([C@H]1CC[C@@]1([C@@H]2CC=C2[C@@]1(C)CC[C@@]1([C@H]2[C@](C)(O)[C@@H](CC1)C)C(=O)O)C)C	
OC[C@]1(C)[C@H](O)[C@H](O)C[C@]2([C@H]1CC[C@@]1([C@@H]2CC=C2[C@@]1(C)CC[C@@]1([C@H]2[C@](C)(O)[C@@H](CC1)C)C(=O)O)C)C	
O[C@@H]1C[C@@]2(C)[C@H](C([C@H]1O)(C)C)CC[C@@]1([C@@H]2CC=C2[C@@]1(C)CC[C@@]1([C@H]2CC(C)(C)CC1)C(=O)O)C	
CC(=O)[C@@H]1CC[C@]2([C@H]1[C@H]1CC[C@H]3[C@@]([C@]1(C)CC2)(C)CC[C@@H]1[C@]3(C)CC[C@@H](C1(C)C)O)C(=O)O	
OC(=O)C[C@@]1(C)[C@@H](CC[C@@]2([C@@H]1CC[C@H]1[C@@]2(C)CC[C@@]2([C@@H]1[C@@H](CC2)C(=O)C)C(=O)O)C)C(C(=O)O)(C)C	
CC(=C)[C@@H]1CC[C@]2([C@H]1[C@H]1CC[C@H]3[C@@]([C@]1(C)CC2)(C)CC[C@@H]1[C@]3(C)C[C@@H](O)[C@@H](C1(C)C)O)C(=O)O	
OC(=O)CC(=O)O[C@@H]1CC[C@]2([C@H](C1(C)C)CCC1=C2C[C@@H](O)[C@]2([C@@]1(C)CC[C@@H]2[C@@H](CC(=O)[C@H]([C@@H](C(=O)O)C)C)C)C)C	
OC(=O)CC(=O)O[C@@H]1CC[C@]2([C@H](C1(C)C)CCC1=C2C[C@H]2O[C@]3(OC(=O)[C@H]([C@@H]3C)C)C[C@H]([C@@H]3[C@@]2([C@@]1(C)CC3)C)C)C	
OC(=O)CC(=O)O[C@@H]1CC[C@]2([C@H](C1(C)C)CCC1=C2C[C@H]2O[C@@]3(OC(=O)[C@H]([C@@H]3C)C)C[C@H]([C@@H]3[C@@]2([C@@]1(C)CC3)C)C)C	(Isaka et al. 2017)
OC(=O)CC(=O)O[C@@H]1CC[C@]2([C@H](C1(C)C)CCC1=C2CC[C@]2([C@@]1(C)CCC2[C@@H](CC(=O)[C@H]([C@@H](C(=O)O)C)C)C)C)C	
O/N=C/1\CC[C@]2(C(C1(C)C)CC[C@@]1(C2CCC2[C@@]1(C)CC[C@@]1(C2[C@@H](CC1)C(=C)C)C(=O)O)C)C	
COC(=O)[C@@H](C(C)C)NC(=O)NC12CC[C@H](C2C2[C@@](CC1)(C)[C@]1(C)CCC3[C@]([C@H]1CC2)(C)CCC(=O)C3(C)C)C(=C)C	
NNC(=O)[C@@]12CC[C@H](C2C2[C@@](CC1)(C)[C@]1(C)CCC3[C@]([C@H]1CC2)(C)CCC(=O)C3(C)C)C(=C)C	
NNC(=O)[C@@]12CC[C@H](C2C2[C@@](CC1)(C)[C@]1(C)CCC3[C@]([C@H]1CC2)(C)CC/C(=N\O)/C3(C)C)C(=C)C	
CSCC[C@@H](C(=O)OC)NC(=O)[C@@]12CC[C@H](C2C2[C@@](CC1)(C)[C@]1(C)CCC3[C@]([C@H]1CC2)(C)CCC(=O)C3(C)C)C(=C)C	
COC(=O)[C@@H](NC(=O)[C@@]12CC[C@H](C2C2[C@@](CC1)(C)[C@]1(C)CCC3[C@]([C@H]1CC2)(C)CCC(=O)C3(C)C)C(=C)C)C	
OCCNC(=O)[C@@]12CC[C@H](C2C2[C@@](CC1)(C)[C@]1(C)CCC3[C@]([C@H]1CC2)(C)CCC(=O)C3(C)C)C(=C)C	
O=C=N[C@@]12CC[C@H](C2C2[C@@](CC1)(C)[C@]1(C)CCC3[C@]([C@H]1CC2)(C)CCC(=O)C3(C)C)C(=C)C	(Baltina et al. 2003)
COC(=O)[C@H](NC(=O)NC12CC[C@H](C2C2[C@@](CC1)(C)[C@]1(C)CCC3[C@]([C@H]1CC2)(C)CCC(=O)C3(C)C)C(=C)C)CC(C)C	
CSCC[C@H](C(=O)OC)NC(=O)NC12CC[C@H](C2C2[C@@](CC1)(C)[C@]1(C)CCC3[C@]([C@H]1CC2)(C)CC/C(=N\O)/C3(C)C)C(=C)C	
CCCCCCCCCCCCCCCCCCNC(=O)[C@@]12CC[C@H](C2C2[C@@](CC1)(C)[C@]1(C)CCC3[C@]([C@H]1CC2)(C)CCC(=O)C3(C)C)C(=C)C	
COC(=O)[C@@H](NC(=O)[C@@]12CC[C@H](C2C2[C@@](CC1)(C)[C@]1(C)CCC3[C@]([C@H]1CC2)(C)CCC(=O)C3(C)C)C(=C)C)CC(C)C	
COC(=O)[C@H](C(C)C)NC(=O)[C@@]12CC[C@H](C2C2[C@@](CC1)(C)[C@]1(C)CCC3[C@]([C@H]1CC2)(C)CCC(=O)C3(C)C)C(=C)C	
CSCC[C@H](C(=O)OC)NC(=O)NC12CC[C@H](C2C2[C@@](CC1)(C)[C@]1(C)CCC3[C@]([C@H]1CC2)(C)CCC(=O)C3(C)C)C(=C)C	
CC(=C)[C@@H]1CC[C@]2(C1C1CC[C@H]3[C@@]([C@@]1(C)CC2)(C)CCC1[C@]3(C)CCC(=O)C1(C)C)C(=O)N	
OC/C(=C/CC[C@H]([C@H]1CC[C@@]2([C@]1(C)CC=C1C2=CC[C@@H]2[C@]1(C)CC[C@@H](C2(C)C)O)C)C)/C	
OC/C(=C/CC[C@H]([C@H]1CC[C@@]2([C@]1(C)CCC1=C2C(=O)C[C@@H]2[C@]1(C)CC[C@@H](C2(C)C)O)C)C)/C	(Mothana et al. 2003)
O=C(C[C@](C1=C[C@@H]([C@@]2([C@]1(C)C(=O)C=C1[C@]32O[C@@H]3C[C@@H]2[C@]1(C)CCC(=O)C2(C)C)C)O)(O)C)CC(C(=O)O)C	
CC(=C)[C@@H]1CC[C@]2([C@H]1[C@H]1CCC3[C@@]([C@]1(C)CC2)(C)CCC1[C@]3(C)CCC(=O)C1(C)C)C(=O)O	(Baltina et al. 2003)
CC(=C)[C@@H]1CC[C@]2([C@H]1[C@H]1CCC3[C@@]([C@]1(C)CC2)(C)CCC1[C@]3(C)CC[C@@H](C1(C)C)O)C(=O)O	

O/N=C/1\CC[C@]2(C(C1(C)C)CC[C@@]1(C2CC[C@H]2[C@@]1(C)CC[C@@]1([C@@H]2[C@@H](CC1)C(=C)C)C(=O)O)C)C	
NNC(=O)[C@@]12CC[C@H]([C@@H]2C2[C@@](CC1)(C)[C@]1(C)CCC3[C@](C1CC2)(C)CCC(=O)C3(C)C)C(=C)C	
NNC(=O)[C@@]12CC[C@H]([C@@H]2C2[C@@](CC1)(C)[C@]1(C)CCC3[C@](C1CC2)(C)CC/C(=N\O)/C3(C)C)C(=C)C	
O=C=N[C@@]12CC[C@H]([C@@H]2C2[C@@](CC1)(C)[C@]1(C)CCC3[C@](C1CC2)(C)CCC(=O)C3(C)C)C(=C)C	
COC(=O)[C@@H](C(C)C)NC(=O)N[C@@]12CC[C@H]([C@@H]2C2[C@@](CC1)(C)[C@]1(C)CCC3[C@](C1CC2)(C)CCC(=O)C3(C)C)C(=C)C	
COC(=O)[C@H](NC(=O)N[C@@]12CC[C@H]([C@@H]2C2[C@@](CC1)(C)[C@]1(C)CCC3[C@](C1CC2)(C)CCC(=O)C3(C)C)C(=C)C)CC(C)C	
CSCC[C@H](C(=O)OC)NC(=O)N[C@@]12CC[C@H]([C@@H]2C2[C@@](CC1)(C)[C@]1(C)CCC3[C@](C1CC2)(C)CCC(=O)C3(C)C)C(=C)C	
CSCC[C@H](C(=O)OC)NC(=O)N[C@@]12CC[C@H]([C@@H]2C2[C@@](CC1)(C)[C@]1(C)CCC3[C@](C1CC2)(C)CC/C(=N\O)/C3(C)C)C(=C)C	
CCCCCCCCCCCCCCCCCCNC(=O)[C@@]12CC[C@H]([C@@H]2C2[C@@](CC1)(C)[C@]1(C)CCC3[C@](C1CC2)(C)CCC(=O)C3(C)C)C(=C)C	
OCCNC(=O)[C@@]12CC[C@H]([C@@H]2C2[C@@](CC1)(C)[C@]1(C)CCC3[C@](C1CC2)(C)CCC(=O)C3(C)C)C(=C)C	
COC(=O)[C@@H](NC(=O)[C@@]12CC[C@H]([C@@H]2C2[C@@](CC1)(C)[C@]1(C)CCC3[C@](C1CC2)(C)CCC(=O)C3(C)C)C(=C)C)C	
COC(=O)[C@H](C(C)C)NC(=O)[C@@]12CC[C@H]([C@@H]2C2[C@@](CC1)(C)[C@]1(C)CCC3[C@](C1CC2)(C)CCC(=O)C3(C)C)C(=C)C	
COC(=O)[C@@H](NC(=O)[C@@]12CC[C@H]([C@@H]2C2[C@@](CC1)(C)[C@]1(C)CCC3[C@](C1CC2)(C)CCC(=O)C3(C)C)C(=C)C)CC(C)C	
CSCC[C@@H](C(=O)OC)NC(=O)[C@@]12CC[C@H]([C@@H]2C2[C@@](CC1)(C)[C@]1(C)CCC3[C@](C1CC2)(C)CCC(=O)C3(C)C)C(=C)C	
CC(=C)[C@@H]1CC[C@]2([C@H]1C1CCC3[C@@]([C@@]1(C)CC2)(C)CCC1[C@]3(C)CCC(=O)C1(C)C)C(=O)N	
OC[C@H]1O[C@@H](O[C@@H]2CCC3=C(C2(C)C)CC[C@@H]2[C@@H](C3)CC[C@]3([C@]2(CC[C@@H]3[C@@H](CCC(=O)C(O)(C)C)C)C(=O)O)C)[C@@H]([C@H]([C@@H]1O)O)O	
C[C@@H]([C@H]1CC[C@@]2([C@]1(C)CC[C@H]1[C@H]2CCC2=C(C1)CC[C@H](C2(C)C)O)C(=O)O)CCC(=O)C(O)(C)C	
C[C@@H]([C@H]1CC[C@@]2([C@]1(C)CC[C@H]1[C@H]2CCC2=C(C1)CC[C@H](C2(C)C)O)C(=O)O)CC[C@H](C(O)(C)C)O	
CC(=O)OCC1O[C@@H](O[C@@H]2CC=C3[C@H](C2(C)C)CC[C@@H]2[C@@H](C3)CC[C@]3([C@]2(CC[C@@H]3[C@@H](CC[C@@H](C(O)(C)C)O[C@H]2OC[C@@H]([C@H](C2O)O)O)C)C(=O)O)C)C([C@@H]([C@@H]1O)O)O	
C[C@@H]([C@H]1CC[C@@]2([C@]1(C)CCC1=C2CC[C@@H]2[C@]1(C)CCC(C2(C)C)O)C(=O)O)CC[C@@H](C(O)(C)C)O	(Lv et al. 2016)
C[C@@H]([C@H]1CC[C@@]2([C@]1(C)CCC1=C2CC[C@@H]2[C@]1(C)CCC(C2(C)C)O)C(=O)O)CC[C@H](C(O)(C)C)O	
CC(=O)OCC1O[C@@H](OC2CC[C@]3([C@H](C2(C)C)CCC2=C3CC[C@]3([C@]2(CC[C@@H]3[C@@H](CC[C@@H](C(O)(C)C)O)C)C(=O)O)C)C)C([C@@H]([C@@H]1O)O)O	
OCC1O[C@H](O[C@H](C(O)(C)C)CC[C@H]([C@H]2CC[C@@]3([C@]2(C)CCC2=C3CC[C@@H]3[C@]2(C)CCC(C3(C)C)O)C(=O)O)C)C([C@@H]([C@@H]1O)O)O	
CC([C@@H](O[C@H]1OC[C@@H]([C@H](C1O)O)O)CC[C@H]([C@H]1CC[C@@]2([C@]1(C)CCC1=C2CC[C@@H]2[C@]1(C)CCC(C2(C)C)O[C@@H]1OC(COC(=O)C)[C@H]([C@H](C1O)O)O)C(=O)O)C)(O)C	
OC[C@]1(C)[C@H](O)[C@H](O)C[C@]2([C@H]1CC[C@@]1([C@@H]2CC=C2[C@@]1(C)CC[C@]13C2=C(C)[C@](CC1)(OO3)C)C)C	
OC[C@@]1(C)[C@H](O)[C@H](O)C[C@]2([C@H]1CC[C@@]1([C@@H]2CC=C2[C@@]1(C)CCC(=C2C(=O)C)CCC(=O)C)C)C	
OC[C@]1(C)[C@@H](O)[C@H](O)C[C@]2([C@H]1CC[C@@]1([C@@H]2CC=C2[C@@]1(C)CCc1c2c(C)c(cc1)C)C)C	
OC[C@@]1(C)[C@H](O)CC[C@]2([C@H]1CC[C@@]1([C@@H]2CC=C2[C@@]1(C)CC[C@@]13C2=C(C)[C@@](CC1)(OO3)C)C)C	(Liu et al. 2018)
OC[C@]1(C)[C@H](O)CC[C@]2([C@H]1CC[C@@]1([C@@H]2CC=C2[C@@]1(C)CC[C@]13C2=C(C)[C@](CC1)(OO3)C)C)C	
OC[C@]1(C)[C@H](O)[C@H](O)[C@H]([C@]2([C@H]1CC[C@@]1([C@@H]2CC=C2[C@@]1(C)CCc1c2c(C)c(cc1)C)C)C)O	

OC[C@@]1(C)[C@H](O)CC[C@]2([C@H]1CC[C@@]1([C@@H]2CC=C2[C@@]1(C)CCc1c2c(C)c(cc1)C)C)C	
OC[C@@]1(C)[C@H](O)[C@H](O)C[C@]2([C@H]1CC[C@@]1([C@@H]2CC=C2[C@@]1(C)CCc1c2c(C)c(cc1)C)C)C	
OC[C@@]1(C)[C@H](O)C[C@H]([C@]2([C@H]1CC[C@@]1([C@@H]2CC=C2[C@@]1(C)CCc1c2c(C)c(cc1)C)C)C)O	
CO[C@@H]1[C@@H](O)[C@H](O[C@@H]2[C@@H](O)[C@H](O[C@@H]3[C@@H](C)O[C@H]([C@@H]([C@H]3O)O)O[C@H]3[C@@H](OC[C@H]([C@@H]3O)OS(=O)(=O)[O-])O[C@H]3CC[C@]4([C@H](C3(C)C)CC=C3[C@@H]4CC[C@]45[C@@]3(C)C[C@@H]([C@@H]5[C@](OC4=O)(C)CCC=C(C)C)OC(=O)C)C)O[C@@H]([C@H]2O)COS(=O)(=O)[O-])O[C@@H]([C@H]1O)COS(=O)(=O)[O-].[Na+].[Na+].[Na+]	(Maier et al. 2001)
CO[C@@H]1[C@@H](O)[C@H](O[C@@H]2[C@@H](O)[C@H](O[C@@H]3[C@@H](C)O[C@H]([C@@H]([C@H]3O)O)O[C@H]3[C@@H](OC[C@H]([C@@H]3O)OS(=O)(=O)[O-])O[C@H]3CC[C@]4([C@H](C3(C)C)CC=C3[C@@H]4CC[C@]45[C@@]3(C)C[C@@H]([C@@H]5[C@](OC4=O)(C)CCCC(C)C)OC(=O)C)C)O[C@@H]([C@H]2O)COS(=O)(=O)[O-])O[C@@H]([C@H]1O)COS(=O)(=O)[O-].[Na+].[Na+].[Na+]	
CO[C@@H]1[C@@H](O)[C@H](O[C@@H]2[C@@H](O)[C@H](O[C@@H]3[C@@H](C)O[C@H]([C@@H]([C@H]3O)O)O[C@H]3[C@@H](OC[C@H]([C@@H]3O)OS(=O)(=O)O)O[C@H]3CC[C@]4([C@H](C3(C)C)CC=C3[C@@H]4CC[C@]45[C@@]3(C)C[C@@H]([C@@H]5[C@](OC4=O)(C)CCC=C(C)C)OC(=O)C)C)O[C@@H]([C@H]2O)COS(=O)(=O)O)O[C@@H]([C@H]1O)COS(=O)(=O)O.[NaH]	
CO[C@@H]1[C@@H](O)[C@H](O[C@@H]2[C@@H](O)[C@H](O[C@@H]3[C@@H](C)O[C@H]([C@@H]([C@H]3O)O)O[C@H]3[C@@H](OC[C@H]([C@@H]3O)OS(=O)(=O)O)O[C@H]3CC[C@]4([C@H](C3(C)C)CC=C3[C@@H]4CC[C@]45[C@@]3(C)C[C@@H]([C@@H]5[C@](OC4=O)(C)CCCC(C)C)OC(=O)C)C)O[C@@H]([C@H]2O)COS(=O)(=O)O)O[C@@H]([C@H]1O)COS(=O)(=O)O.[NaH]	
CO[C@@H]1CO[C@H]([C@@H]([C@H]1O)O)OCC[C@H](C(C)C)CC[C@H]([C@H]1[C@@H](O)[C@H]([C@@H]2[C@]1(C)CC[C@H]1[C@@]2(O)C[C@@H]([C@@H]2[C@]1(C)CC[C@@H]([C@@H]2O)O)OS(=O)(=O)[O-])O)C.[Na+]	(Wang et al. 2002)
OC[C@H]1O[C@H]([C@@H]([C@H]1O)O[C@@H]1OC[C@H]([C@@H]([C@H]1OC)O)OC)O[C@H](C(C)C)CC[C@H]([C@H]1C[C@H]([C@@H]2[C@]1(C)CC[C@H]1[C@@]2(O)C[C@@H]([C@@H]2[C@]1(C)CC[C@@H]([C@@H]2O)O)OS(=O)(=O)[O-])O)C.[Na+]	
OC[C@H]1O[C@H]([C@@H]([C@H]1O)O[C@@H]1OC[C@H]([C@@H]([C@H]1OC)O)OC)O[C@H](C(C)C)CC[C@H]([C@H]1C[C@H]([C@@H]2[C@]1(C)CC[C@H]1[C@H]2C[C@@H]([C@@H]2[C@]1(C)CC[C@@H]([C@@H]2O)O)O)O)C	
CO[C@H]1[C@@H](OC[C@H]([C@@H]1O)O)O[C@H]1[C@H](OCC(C(=C)CC[C@H]([C@H]2C[C@H]([C@@H]3[C@]2(C)CC[C@H]2[C@@]3(O)C[C@@H]([C@@H]3[C@]2(C)CC[C@@H]([C@@H]3O)O)O)O)C)C)OC[C@H]([C@@H]1OS(=O)(=O)[O-])O.[Na+]	
OC[C@H]1O[C@H]([C@@H]([C@H]1O)O[C@@H]1OC[C@H]([C@@H]([C@H]1OC)O)OC)O[C@H](C(C)C)CC[C@H]([C@H]1C[C@H]([C@@H]2[C@]1(C)CC[C@H]1[C@@]2(O)C[C@@H]([C@@H]2[C@]1(C)CC[C@@H]([C@@H]2O)O)O)O)C	
CO[C@H]1[C@@H](OC[C@H]([C@@H]1O)O)O[C@H]1[C@H](OC[C@H]([C@@H](/C=C/[C@H]([C@H]2C[C@H]([C@@H]3[C@]2(C)CC[C@H]2[C@@]3(O)C[C@@H]([C@@H]3[C@]2(C)CC[C@@H]([C@@H]3O)O)O)O)C)C)C)OC[C@H]([C@@H]1OS(=O)(=O)[O-])O.[Na+]	
CO[C@@H]1CO[C@H]([C@@H]([C@H]1O)O)OCC[C@H](C(C)C)CC[C@H]([C@H]1[C@@H](O)[C@H]([C@@H]2[C@]1(C)CC[C@H]1[C@H]2C[C@@H]([C@@H]2[C@]1(C)CC[C@@H]([C@@H]2O)O)OS(=O)(=O)[O-])O)C.[Na+]	
CO[C@H]1[C@@H](OC[C@H]([C@@H]1O)O)O[C@H]1[C@H](OCC(C(=C)CC[C@H]([C@H]2C[C@H]([C@@H]3[C@]2(C)CC[C@H]2[C@@]3(O)C[C@@H]([C@@H]3[C@]2(C)CC[C@@H]([C@@H]3O)O)O)O)C)C)OC[C@H]([C@@H]1O)O	
CO[C@H]1[C@@H](OC[C@H]([C@@H]1O)O)O[C@H]1[C@@H](OC[C@H]([C@@H]1OS(=O)(=O)[O-])O)OCC(C(=C)CC[C@H]([C@H]1C[C@H]([C@@H]2[C@]1(C)CC[C@H]1[C@@]2(O)C[C@@H]([C@@H]2[C@]1(C)CC[C@@H](C2)O)O)O)C)C.[Na+]	
CO[C@H]1[C@@H](OC[C@H]([C@@H]1O)O)O[C@H]1[C@H](OCC(C(=C)CC[C@H]([C@H]2C[C@H]([C@@H]3[C@]2(C)CC[C@H]2[C@@]3(O)C[C@@H]([C@@H]3[C@]2(C)CC[C@@H]([C@@H]3O)O)O)O)C)C)OC[C@H]([C@@H]1OS(=O)(=O)O)O.[NaH]	
CO[C@H]1[C@@H](OC[C@H]([C@@H]1O)O)O[C@H]1[C@@H](OC[C@H]([C@@H]1OS(=O)(=O)O)O)OCC(C(=C)CC[C@H]([C@H]1C[C@H]([C@@H]2[C@]1(C)CC[C@H]1[C@@]2(O)C[C@@H]([C@@H]2[C@]1(C)CC[C@@H](C2)O)O)O)C)C.[NaH]	
CO[C@H]1[C@@H](OC[C@H]([C@@H]1O)O)O[C@H]1[C@H](OC[C@H]([C@@H](/C=C/[C@H]([C@H]2C[C@H]([C@@H]3[C@]2(C)CC[C@H]2[C@@]3(O)C[C@@H]([C@@H]3[C@]2(C)CC[C@@H]([C@@H]3O)O)O)O)C)C)C)OC[C@H]([C@@H]1OS(=O)(=O)O)O.[NaH]	

OC[C@@H]1O[C@H]([C@@H]([C@H]1O)O[C@@H]1OC[C@H]([C@@H]([C@H]1OC)O)OC)O[C@H](C(C)C)CC[C@H]([C@H]1C[C@H]([C@@H]2[C@]1(C)CC[C@H]1[C@@]2(O)C[C@@H]([C@@H]2[C@]1(C)CC[C@@H]([C@@H]2O)O)O)O)C	
OC[C@H]1O[C@H]([C@@H]([C@H]1O)O[C@@H]1OC[C@H]([C@@H]([C@H]1OC)O)OC)O[C@H](C(C)C)CC[C@H]([C@H]1C[C@H]([C@@H]2[C@]1(C)CC[C@H]1[C@@]2(O)C[C@@H]([C@@H]2[C@]1(C)CC[C@@H]([C@@H]2O)O)OS(=O)(=O)O)O)C.[NaH]	
CO[C@@H]1CO[C@H]([C@@H]([C@H]1O)O)OCC[C@H](C(C)C)CC[C@H]([C@H]1[C@@H](O)[C@H]([C@@H]2[C@]1(C)CC[C@H]1[C@@]2(O)C[C@@H]([C@@H]2[C@]1(C)CC[C@@H]([C@@H]2O)O)OS(=O)(=O)O)O)C.[NaH]	
CO[C@@H]1CO[C@H]([C@@H]([C@H]1O)O)OCC[C@H](C(C)C)CC[C@H]([C@H]1[C@@H](O)[C@H]([C@@H]2[C@]1(C)CC[C@H]1[C@H]2C[C@@H]([C@@H]2[C@]1(C)CC[C@@H]([C@@H]2O)O)OS(=O)(=O)O)O)C.[NaH]	
O=C1CC[C@]2([C@H](C1(C)C)CC[C@@]1([C@@H]2CC[C@H]2[C@@]1(C)CCC1[C@]2(C)CC[C@@H]1C(O)(C)C)C)C	
O[C@H]1C[C@@H]2[C@@]3(C)CCC(=O)C([C@@H]3CC[C@]2([C@]2([C@@]31OC(=O)[C@]1([C@H]3[C@@H](C)C(C)(C)CC1)CC2)C)C)(C)C	
CC(=CCC[C@]([C@H]1CC[C@@]2([C@@H]1CC[C@H]1[C@@]2(C)CC[C@@H]2[C@]1(C)CCC(=O)C2(C)C)C)(O)C)C	
CC(=CCC[C@@]([C@H]1CC[C@@]2([C@@H]1CC[C@H]1[C@@]2(C)CC[C@@H]2[C@]1(C)CC[C@@H](C2(C)C)O)C)(O)C)C	
OC(=O)CC[C@@]1(C)[C@@H](CC[C@@]2([C@@H]1CC[C@H]1[C@@]2(C)CC[C@@H]1[C@]1(C)CC[C@@H](O1)C(O)(C)C)C)C(=C)C	(Poehland et al. 1987)
OC(=O)CC[C@@]1(C)[C@@H](CC[C@@]2([C@@H]1CC[C@H]1[C@@]2(C)CC[C@@H]1[C@]1(C)CC[C@H](O1)C(O)(C)C)C)C(=C)C	
C[C@@H]1CC[C@]2([C@@H]([C@H]1C)C1=CC[C@H]3[C@@]([C@@]1(CC2)C)(C)CC[C@@H]1[C@]3(C)CCC(=O)C1(C)C)C(=O)O	
CC(=CCCC(=C)[C@H]1CC[C@@]2([C@@H]1CC[C@H]1[C@@]2(C)CC[C@@H]2[C@]1(C)CC[C@@H](C2(C)C)O)C)C	
OC(=O)CC[C@@]1(C)[C@@H](CC[C@@]2([C@@H]1CC[C@H]1[C@@]2(C)CC[C@@H]1[C@](CCC=C(C)C)(O)C)C)C(=C)C	
OC[C@H]1O[C@@H](O[C@H]2[C@@H](OC[C@@H]([C@@H]2O)O[C@@H]2O[C@H](CO)[C@H]([C@@H]([C@H]2O[C@@H]2O[C@@H](C)[C@@H]([C@H]([C@H]2O)O)O)O)O)O[C@H]2CC[C@]3([C@H](C2(C)C)CC[C@@]2([C@@H]3CC[C@]34[C@@]2(C)C[C@H]([C@@]2([C@H]4CC(C)(C)CC2)CO3)O)C)C)[C@@H]([C@H]([C@@H]1O)O)O	
OC[C@H]1O[C@@H](O[C@H]2[C@@H](OC[C@@H]([C@@H]2O)O[C@@H]2O[C@H](CO)[C@H]([C@@H]([C@H]2O[C@@H]2O[C@@H](C)[C@@H]([C@H]([C@H]2O)O)O)O)O)O[C@H]2CC[C@]3([C@H](C2(C)C)CC[C@@]2([C@@H]3CC[C@]34[C@@]2(C)C[C@H]([C@@]2([C@H]4C[C@@](C)(C=O)CC2)CO3)O)C)C)[C@@H]([C@H]([C@@H]1O)O)O	
OC[C@H]1O[C@@H](O[C@H]2[C@@H](OC[C@@H]([C@@H]2O)O[C@@H]2O[C@H](CO)[C@H]([C@@H]([C@H]2O[C@@H]2O[C@@H](C)[C@@H]([C@H]([C@H]2O)O)O)O)O)O[C@H]2CC[C@]3([C@H](C2(C)C)CC[C@@]2([C@@H]3CC[C@]34[C@@]2(C)C[C@H]([C@@]2([C@H]4CC(C)(C)CC2)C(=O)O3)O)C)C)[C@@H]([C@H]([C@@H]1O)O)O	
OC[C@H]1O[C@@H](O[C@H]2[C@@H](OC[C@@H]([C@@H]2O)O[C@@H]2O[C@H](CO)[C@H]([C@@H]([C@H]2O[C@@H]2OC[C@H]([C@@H]([C@H]2O)O)O)O)O)O[C@H]2CC[C@]3([C@H](C2(C)C)CC[C@@]2([C@@H]3CC[C@]34[C@@]2(C)C[C@H]([C@@]2([C@H]4C[C@@](C)(C=O)CC2)CO3)O)C)C)[C@@H]([C@H]([C@@H]1O)O)O	
OC[C@H]1O[C@@H](O[C@H]2[C@@H](OC[C@@H]([C@@H]2O)O[C@@H]2O[C@H](CO)[C@H]([C@@H]([C@H]2O[C@@H]2OC[C@H]([C@@H]([C@H]2O)O)O)O)O)O[C@H]2CC[C@]3([C@H](C2(C)C)CC[C@@]2([C@@H]3CC[C@]34[C@@]2(C)C[C@H]([C@@]2([C@H]4CC(C)(C)CC2)CO3)O)C)C)[C@@H]([C@H]([C@@H]1O)O)O	(Bloor and Qi 1994)
OC[C@H]1O[C@@H](O[C@H]2[C@@H](OC[C@@H]([C@@H]2O)O[C@@H]2O[C@H](CO)[C@H]([C@@H]([C@H]2O[C@@H]2OC[C@H]([C@@H]([C@H]2O)O)O)O)O)O[C@H]2CC[C@]3([C@H](C2(C)C)CC[C@@]2([C@@H]3CC[C@]34[C@@]2(C)C[C@H]([C@@]2([C@H]4CC(C)(C)CC2)C(=O)O3)O)C)C)[C@@H]([C@H]([C@@H]1O)O)O	
OC[C@H]1O[C@@H](O[C@H]2[C@@H](OC[C@@H]([C@@H]2O)O[C@@H]2O[C@H](CO)[C@H]([C@@H]([C@H]2O[C@@H]2O[C@@H](C)[C@@H]([C@H]([C@H]2O)O)O)O)O)O[C@H]2CC[C@]3([C@H](C2(C)C)CC[C@@]2([C@@H]3CC[C@]34[C@@]2(C)C[C@H]([C@@]2([C@H]4CC(C)(C)CC2)C(O3)O)O)C)C)[C@@H]([C@H]([C@@H]1O)O)O	
OC[C@H]1O[C@@H](O[C@H]2[C@@H](OC[C@@H]([C@@H]2O)O[C@@H]2O[C@H](CO)[C@H]([C@@H]([C@H]2O[C@@H]2OC[C@H]([C@@H]([C@H]2O)O)O)O)O)O[C@H]2CC[C@]3([C@H](C2(C)C)CC[C@@]2([C@@H]3CC[C@]34[C@@]2(C)C[C@H]([C@@]2([C@H]4CC(C)(C)CC2)C(O3)O)O)C)C)[C@@H]([C@H]([C@@H]1O)O)O	

O=C1O[C@]23[C@](C1)(O)OC([C@@H]3C[C@@H]([C@@H]1[C@@]3(C2)CC[C@]2([C@]4([C@](O3)(C1=O)O[C@H]1[C@@H]([C@H]4[C@H](C2=O)C)OC(=O)[C@]1(C)O)C)C)O)(C)C	(Cheng et al. 2010)
O=C1O[C@]23[C@](C1)(O)OC([C@@H]3C[C@@H]([C@@H]1[C@@]3(C2)CC[C@]2([C@]4([C@](O3)(C1=O)O[C@@H]1[C@H](C)C(=O)O[C@@H]1[C@H]4[C@H](C2=O)C)C)C)O)(C)C	
O=C1C[C@H]([C@H](O1)[C@@H](/C=C\1/C=C(C(=O)O1)C)C)[C@H]1C[C@]2([C@@H]([C@H]1O)CC[C@@H]1[C@@]3(C2)OC(=O)C[C@@]3(O)OC1(C)C)O	
O=C1O[C@]23[C@](C1)(O)OC([C@@H]3C[C@@H]([C@@H]1[C@@]3(C2)CC[C@]2([C@H]4[C@](O3)(C1=O)O[C@H]1[C@@H]([C@H]4[C@H](C2=O)C)OC(=O)[C@]1(C)O)C)O)(C)C	
O=C1C[C@@H]2[C@@]3(O1)[C@@H](O)[C@]14CC[C@]5([C@@H]([C@](O1)(C(=O)[C@@H]4CC[C@H]3C(O2)(C)C)O)[C@H]5[C@@H]([C@@H]([C@@H]1C[C@H](C(=O)O1)C)O)C)C	
O=C1O[C@@H](C[C@H]1C)[C@H]([C@H]([C@@H]1[C@H]2[C@]1(C)CC[C@@]13O[C@]2(O)C(=O)[C@@H]1CC[C@@H]1[C@@]2([C@H]3O)OC(=O)[C@@H]([C@H]2OC1(C)C)O)C)O	
O=C1O[C@]23[C@](C1)(O)OC([C@@H]3C[C@@H]([C@@H]1[C@@]3(C2)CC[C@]2([C@H]4[C@](O3)(C1=O)O[C@@H]1[C@H](C)C(=O)O[C@@H]1[C@H]4[C@@H](C2=O)C)C)O)(C)C	
O=C1O[C@]23[C@](C1)(O)OC([C@@H]3C[C@@H]([C@@H]1[C@@]3(C2)CC[C@]2([C@H]4[C@](O3)(C1=O)O[C@@H]1[C@H](C)C(=O)O[C@@H]1[C@H]4[C@H](C2=O)C)C)O)(C)C	
CC(=O)O[C@@H]([C@H]([C@@H]1[C@H]2[C@]1(C)CC[C@@]13O[C@]2(O)C(=O)[C@@H]1CC[C@@H]1[C@@]2([C@H]3O)OC(=O)C[C@H]2OC1(C)C)C)[C@@H]1C=C(C(=O)O1)C	
CC(=O)O[C@@H]([C@H]([C@@H]1[C@H]2[C@]1(C)CC[C@@]13O[C@]2(O)C(=O)[C@@H]1CC[C@@H]1[C@@]2([C@@H]3O)OC(=O)C[C@H]2OC1(C)C)C)[C@@H]1C[C@H](C(=O)O1)C	
COC(=O)C1(O)OCC23C1C(C)(C(C1C3C(C(CC2OC(=O)C=Cc2ccccc2)O)(C)CO1)O)C12OC2(C)C2CC1OC1C2(O)C=CO1	
C[C@@H]1CC[C@]2([C@@H]([C@H]1C)[C@]1(C)CC[C@@]3([C@@](C1=CC2)(C)[C@@H](O)C[C@@]1([C@@H](C3=O)C[C@@H](C1(C)C)O)O)C)C	(Liu et al. 2017)
C[C@@H]1CC[C@]2([C@@H]([C@H]1C)[C@]1(C)CC[C@@]3([C@@](C1=CC2=O)(C)[C@@H](O)C[C@@]1([C@@H](C3=O)C[C@@H](C1(C)C)O)O)C)C	
O[C@H]1C[C@]2(C)C[C@@H](O)[C@]3([C@H]([C@@H]2[C@H]([C@@H]1C)C)CC=C1C3=CC=C2[C@]1(C)CCC(=O)C2(C)C)C	
COC(=O)[C@@]12CC[C@H]([C@@H]2[C@@H]2[C@](CC1)(C)[C@]1(C)CC[C@@H]3[C@]([C@H]1CC2)(C)c1c(=O)c2ccccc2[nH]c1C3(C)C)C(=O)C	(Khusnutdinova et al. 2019)
O=c1c2ccccc2[nH]c2c1[C@@]1(C)[C@H](C2(C)C)CC[C@@]2([C@@H]1CC[C@H]1[C@@]2(C)CC[C@]23[C@@H]1[C@@H](OC3=O)C(C)(C)CC2)C	
OC[C@H]1O[C@@H](O[C@H]2CC[C@]3([C@H](C2(C)C)CC[C@@]2([C@@H]3C[C@@H](O)[C@H]3[C@@]2(C)CC[C@@H]3[C@](CCC=C(C)C)(O)C)C)C)[C@@H]([C@H]([C@@H]1O)O)O[C@@H]1O[C@H](CO)[C@H]([C@@H]([C@H]1O[C@@H]1OC[C@H]([C@@H]([C@H]1O)O)O)O)O)O	(Pei et al. 2011)
C[C@@H]1C[C@@H]2[C@@](CC1=O)(C)CC[C@]1([C@@]2(C)CC=C2[C@@]1(C)C=Cc1c2cc(O)c(c1C)O)C	(Sotanaphun et al. 2005)
C[C@@H]1C[C@@H]2[C@@]([C@H](C1=O)O)(C)CC[C@]1([C@@]2(C)CC=C2[C@@]1(C)C=Cc1c2cc(O)c(c1C)O)C	
Oc1cc2c(c(c1O)C)C=C[C@@]1(C2=CC[C@@]2([C@]1(C)CC[C@@]1([C@H]2C[C@](C)(O)C(=O)C1)C)C)C	
CC1=C2CC[C@@]3([C@]([C@@H]4C2C(C=C1)C(=C(C4=O)O)C)(C)CC[C@@]1([C@H]3C[C@@](C)(O)C(=O)C1)C)C	
CC1=C[C@@H]2[C@@](CC1=O)(C)CC[C@]1([C@@]2(C)CC=C2[C@@]1(C)C=Cc1c2cc(O)c(c1C)O)C	
CC(=O)C[C@H]1C=C2C(=CC[C@@]3([C@]2(C)CC[C@@]2([C@H]3C[C@H](C(=O)C2)C)C)C)c2c1c(C)c(c(c2)O)O	
CC(=O)C[C@H]1C=C2C(=CC[C@@]3([C@]2(C)CC[C@@]2([C@H]3C[C@H](C(=O)[C@@H]2O)C)C)C)c2c1c(C)c(c(c2)O)O	
CC(=O)C[C@H]1C=C2C(=CC[C@@]3([C@]2(C)CC[C@@]2([C@H]3C[C@](C(=O)C2)(C)O)C)C)c2c1c(C)c(c(c2)O)O	
CC(=O)/C=c\1/cc2c(=CC[C@@]3([C@]2(C)CC[C@@]2([C@H]3C[C@@H](C(=O)[C@@H]2O)C)C)C)c2c1c(C)c(O)c(c2)O	
CC(=C)[C@@H]1CC[C@]2([C@H]1[C@H]1CC[C@H]3[C@@]([C@]1(C)CC2)(C)CC[C@@H]1[C@]3(C)CCC(=O)C1(C)C)C	(Madureira et al. 2003)
CC(=O)O[C@H]1CC[C@]2([C@H](C1(C)C)CC[C@@]1([C@@H]2CC[C@H]2[C@@]1(C)CCC1=CCC[C@H]([C@H]21)C(=C)C)C)C	(Flekhter et al. 2007)
CC(=C)[C@@H]1CCC=C2[C@H]1[C@H]1CC[C@H]3[C@@]([C@@]1(CC2)C)(C)CC[C@@H]1[C@]3(C)CC[C@@H](C1(C)C)O	
CC(=O)O[C@H]1CC[C@]2([C@H](C1(C)C)CC[C@@]1([C@@H]2CC[C@H]2[C@@]1(C)CC[C@@]13[C@@H]2C2CCC(O1)O[C@]2(O3)C)C)C	

N#CC[C@@]1(C)C(CC[C@@]2(C1CC[C@H]1[C@@]2(C)CC[C@@]2([C@@H]1[C@@H](CC2)C(=C)C)C(=O)NCCC(=O)OC)C)C(C=O)(C)C	(Tolmacheva et al. 2013)
N#CC[C@@]1(C)C(CC[C@@]2(C1CC[C@H]1[C@@]2(C)CC[C@@]2([C@@H]1[C@@H](CC2)C(=C)C)C(=O)Nc1nccs1)C)C(C=O)(C)C	
CCOC(=O)CNC(=O)C(C1CC[C@@]2(C([C@@]1(C)CC#N)CC[C@H]1[C@@]2(C)CC[C@@]2([C@@H]1[C@@H](CC2)C(=C)C)C(=O)OC)C)(C)C	
N#CC[C@@]1(C)C(CC[C@@]2(C1CC[C@H]1[C@@]2(C)CC[C@@]2([C@@H]1[C@@H](CC2)C(=C)C)C(=O)OC)C)C(C(=O)Nc1nccs1)(C)C	
CCCOC(=O)CCNC(=O)C(C1CC[C@@]2(C([C@@]1(C)CC#N)CC[C@H]1[C@@]2(C)CC[C@@]2([C@@H]1[C@@H](CC2)C(=C)C)C(=O)NCCC(=O)OCC)C)(C)C	
OC[C@]12CC[C@@H](CC1=CC[C@@H]1[C@@H]2CC[C@]2([C@H]1CC[C@@H]2[C@@H](OC(=O)C)C)C)OC(=O)C	(Petrera et al. 2003)
O=C[C@]12CC[C@@H](CC1=CC[C@@H]1[C@@H]2CC[C@]2([C@H]1CC[C@@H]2[C@@H](OC(=O)C)C)C)OC(=O)C	
CO/C=C/[C@]12CC[C@@H](CC1=CC[C@@H]1[C@@H]2CC[C@]2([C@H]1CC[C@@H]2[C@@H](OC(=O)C)C)C)O	
CO/C=C/[C@]12CC[C@@H](CC1=CC[C@@H]1[C@@H]2CC[C@]2([C@H]1CC[C@@H]2[C@@H](OC(=O)C)C)C)OC(=O)C	
CC(=O)O[C@H]1CC[C@]23[C@@](C1)(Cl)C(C[C@@H]1[C@@H]3CC[C@]3([C@H]1CC[C@@H]3[C@@H](OC(=O)C)C)C)C[C@@H]2O	
CC(=O)O[C@H]1CC[C@]23[C@@](C1)(Cl)C(CC2)C[C@@H]1[C@@H]3CC[C@]2([C@H]1CC[C@@H]2[C@@H](OC(=O)C)C)C	
O=C1CC[C@]23C(=C1)C(CC2)C[C@@H]1[C@@H]3CC[C@]2([C@H]1CC[C@@H]2C(=O)C)C	
C/C=C(/C(=O)O[C@H]1[C@H](OC(=O)/C(=C/C)/C)C(C)(C)C[C@@H]2[C@@]1(CO)[C@H](O)C[C@@]1(C2=CCC2[C@@]1(C)CCC1[C@]2(C)CC[C@@H](C1(C)C)O)C)\C	(Yoneda et al. 2018)
C/C=C(/C(=O)O[C@H]1[C@H](OC(=O)/C(=C/C)/C)C(C)(C)C[C@@H]2[C@@]1(CO)[C@H](O)[C@H](O)[C@@]1(C2=CCC2[C@@]1(C)CCC1[C@]2(C)CC[C@@H](C1(C)C)O)C)\C	
C/C=C(/C(=O)O[C@H]1[C@H](O)[C@]2(COC(=O)C)[C@H](O)C[C@@]3(C(=CCC4[C@@]3(C)CCC3[C@]4(C)CC[C@@H](C3(C)C)O)[C@@H]2CC1(C)C)C)\C	
C/C=C(/C(=O)O[C@H]1[C@H](O)[C@]2(CO)[C@H](O)C[C@@]3(C(=CC[C@H]4[C@@]3(C)CC[C@@H]3[C@]4(C)CC[C@@H](C3(C)C)O)[C@@H]2CC1(C)C)C)\C	
OC[C@]12[C@H](O)C[C@@]3(C(=CCC4[C@@]3(C)CCC3[C@]4(C)CC[C@@H](C3(C)C)O)[C@@H]2CC([C@H]([C@@H]1O)O)(C)C)C	
OC[C@@]1(C)[C@H](CC[C@]2([C@H]1CC[C@@]1([C@@H]2CC=C2[C@@]1(C)CC[C@@]1([C@H]2CC(C[C@H]1O)(C)C)C)C)C)O[C@@H]1O[C@H](C(=O)O)[C@H]([C@@H]([C@H]1O[C@@H]1OC[C@@H]([C@@H]([C@H]1O[C@@H]1O[C@@H](C)[C@@H]([C@H]([C@H]1O)O)O)O)O)O)O	(Kinjo et al. 2000)
OC[C@H]1O[C@@H](O[C@H]2[C@@H](O[C@@H]([C@H]([C@@H]2O)O)C(=O)[O-])O[C@H]2CC[C@]3([C@H]([C@@]2(C)CO)CC[C@@]2([C@@H]3CC=C3[C@@]2(C)CC[C@@]2([C@H]3CC(C)(C)C[C@H]2O)C)C)C)[C@@H]([C@H]([C@H]1O)O)O[C@@H]1O[C@@H](C)[C@H]([C@@H]([C@H]1O)O)O	
OC[C@H]1O[C@@H](O[C@H]2C[C@@H]3[C@]4([C@@]5([C@@H]2C(C)(C)[C@H](CC5)O[C@@H]2OC[C@H]([C@@H]([C@H]2OC(=O)C)O)O)C4)CC[C@]2([C@@]3(C)C[C@@H]([C@@H]2[C@@]2(C)CC[C@H](O2)C(O)(C)C)O)C)[C@@H]([C@H]([C@@H]1O)O)O	
OCC1OC(OC2C(OC(C(C2O)O)C(=O)O)OC2CCC3(C(C2(C)C)CCC2(C3CC=C3C2(C)CCC2(C3CC(C)(C)CC2O)C)C)C)C(C(C1O)O)OC1OC(C)C(C(C1O)O)O	
O[C@@H]1[C@@H](O[C@@H]2OC[C@@H]([C@@H]([C@H]2O[C@@H]2O[C@@H](C)[C@@H]([C@H]([C@H]2O)O)O)O)O)[C@@H](O[C@@H]([C@H]1O)C(=O)O)O[C@H]1CC[C@]2([C@H](C1(C)C)CC[C@@]1([C@@H]2CC=C2[C@@]1(C)CC[C@@]1([C@H]2CC(C)(C)C[C@H]1O)C)C)C	
OC[C@H]1O[C@@H](O[C@H]2[C@@H](O[C@@H]([C@H]([C@@H]2O)O)C(=O)O)O[C@H]2CC[C@]3([C@H]([C@@]2(C)CO)CC[C@@]2([C@@H]3CC=C3[C@@]2(C)CC[C@@]2([C@H]3CC(C)(C)C[C@H]2O)C)C)C)[C@@H]([C@H]([C@H]1O)O)O	
OC[C@H]1O[C@@H](O[C@H]2[C@@H](O[C@@H]([C@H]([C@@H]2O)O)C(=O)O)O[C@H]2CC[C@]3([C@H](C2(C)C)CC[C@@]2([C@@H]3CC=C3[C@@]2(C)CC[C@@]2([C@H]3CC(C)(C)C[C@H]2O)C)C)C)[C@@H]([C@H]([C@H]1O)O)O	
OC[C@H]1O[C@@H](O[C@H]2[C@@H](O[C@@H]([C@H]([C@@H]2O)O)C(=O)O)O[C@H]2CC[C@]3([C@H]([C@@]2(C)CO)CC[C@@]2([C@@H]3CC=C3[C@@]2(C)CC[C@@]2([C@H]3CC(C)(C)C[C@H]2O)C)C)C)[C@@H]([C@H]([C@@H]1O)O)O	
O[C@@H]1[C@@H](O)[C@@H](O[C@@H]([C@H]1O)C(=O)O)O[C@H]1CC[C@]2([C@H](C1(C)C)CC[C@@]1([C@@H]2CC=C2[C@@]1(C)CC[C@@]1([C@H]2CC(C)(C)C[C@H]1O)C)C)C	
OC[C@H]1O[C@@H](O[C@H]2[C@@H](O[C@@H]([C@H]([C@@H]2O)O)C(=O)O)O[C@H]2CC[C@]3([C@H]([C@@]2(C)CO)CC[C@@]2([C@@H]3CC=C3[C@@]2(C)CC[C@@]2([C@H]3CC(C)(C)CC2=O)C)C)C)[C@@H]([C@H]([C@H]1O)O)O[C@@H]1O[C@@H](C)[C@@H]([C@H]([C@H]1O)O)O	

O=C(CN1C(=O)CC(C1=O)NC(=O)OCc1ccccc1)NCC(=O)Oc1ccc(cc1)[N+](=O)[O-]	
OC[C@@]1(C)[C@@H](O)CC[C@]2([C@H]1CC[C@@]1([C@@H]2CC=C2[C@@]1(C)CC[C@@]1([C@H]2CC(C[C@H]1O)(C)C)C)C)C	
O[C@H]1CC[C@]2([C@H](C1(C)C)CC[C@@]1([C@@H]2CC=C2[C@@]1(C)CC[C@@]1([C@H]2CC(C)(C)C[C@H]1O)C)C)C	
OC[C@@]1(C)[C@@H](O)CC[C@]2([C@H]1CC[C@@]1([C@@H]2CC=C2[C@@]1(C)CC[C@@]1([C@H]2CC(CC1=O)(C)C)C)C)C	
COC(=O)[C@@]1(C)CC[C@]2([C@H](C1)C1=CC(=O)[C@H]3[C@@]([C@@]1(CC2)C)(C)CCC1[C@]3(C)CC[C@@H](C1(C)C)O)C	(Ikeda et al. 2005)
OC[C@@]1(C)[C@@H](O)CC[C@]2([C@H]1CC[C@@]1([C@@H]2CC=C2[C@@]1(C)CC[C@@]1([C@H]2CC([C@H]([C@H]1O)O)(C)C)C)C)C	
OC[C@@]1(C)C[C@H]2C3=CC[C@H]4[C@@]([C@]3(C)CC[C@]2([C@@H]([C@@H]1O)O)C)(C)CC[C@@H]1[C@]4(C)CC[C@@H]([C@]1(C)CO)O	
OC[C@@]1(C)[C@@H](O)CC[C@]2([C@H]1CC[C@@]1([C@@H]2CC=C2[C@@]1(C)CC[C@@]1([C@H]2C[C@]([C@H]([C@H]1O)O)(C)C(=O)O)C)C)C	
COC(=O)[C@@]1(C)C[C@H]2C3=CC[C@H]4[C@@]([C@]3(C)CC[C@]2([C@@H]([C@@H]1O)O)C)(C)CC[C@@H]1[C@]4(C)CC[C@@H]([C@]1(C)CO)O	
OC[C@@]1(C)C[C@H]2C3=CC[C@H]4[C@@]([C@]3(C)CC[C@]2([C@@H]([C@@H]1O)O)C)(C)CC[C@@H]1[C@]4(C)CC[C@@H](C1(C)C)O	
OC[C@@]1(C)C[C@@H](O)[C@]2([C@@H](C1)C1=CC[C@H]3[C@@]([C@@]1(CC2)C)(C)CC[C@@H]1[C@]3(C)CC[C@@H]([C@@]1(C)CO)O)C	
OC[C@]1(C)C[C@@H](O)[C@]2([C@@H](C1)C1=CC[C@H]3[C@@]([C@@]1(CC2)C)(C)CC[C@@H]1[C@]3(C)CC[C@@H]([C@]1(C)CO)O)C	
OC[C@@]1(C)C[C@@H](O)[C@]2([C@@H](C1)C1=CC[C@H]3[C@@]([C@@]1(CC2)C)(C)CC[C@@H]1[C@]3(C)CC[C@@H](C1(C)C)O)C	
OC[C@@]1(C)[C@@H](O)CC[C@]2([C@H]1CC[C@@]1([C@@H]2CC=C2[C@@]1(C)CC[C@@]1([C@H]2CC([C@H](C1)O)(C)C)C)C)C	
OC[C@@]1(C)[C@@H](O)CC[C@]2([C@H]1CC[C@@]1([C@@H]2CC=C2[C@@]1(C)CC[C@@]1([C@H]2CC(C=C1)(C)C)C)C)C	
OC[C@]1(C)[C@@H](O)CC[C@]2([C@H]1CC[C@@]1([C@@H]2CC=C2[C@@]1(C)CC[C@@]1([C@H]2CC(CC1)(C)C)C(=O)O)C)C	
OC[C@H]1O[C@@H](O[C@H]2[C@@H](O[C@@H]([C@H]([C@@H]2O)O)C(=O)O)O[C@H]2CC[C@]3([C@H]([C@@]2(C)CO)CC[C@@]2([C@@H]3CC=C3[C@@]2(C)CC[C@@]2([C@H]3CC(C)(C)C[C@H]2O)C)C)C)[C@@H]([C@H]([C@H]1O)O)O[C@@H]1O[C@@H](C)[C@@H]([C@H]([C@H]1O)O)O	
O[C@H]1CC[C@]2([C@H](C1(C)C)CC[C@@]1([C@@H]2CC=C2[C@@]1(C)CC[C@@]1([C@H]2CC(C)(C)CC1)C(=O)O)C)C	
O[C@@H]1[C@@H](O[C@@H]2O[C@H](C(=O)O)[C@H]([C@@H]([C@H]2O)O)O)[C@H](O[C@@H]([C@H]1O)C(=O)O)O[C@H]1CC[C@]2([C@H](C1(C)C)CC[C@@]1([C@@H]2C(=O)C=C2[C@@]1(C)CC[C@@]1([C@H]2C[C@](C)(CC1)C(=O)O)C)C)C	
O=C1C=C2[C@@H]3C[C@](C)(CC[C@]3(C)CC[C@]2([C@]2([C@H]1[C@@]1(C)CC[C@@H](C([C@@H]1CC2)(C)C)O)C)C)C(=O)O	
OC[C@@]1(C)[C@H](CC[C@]2([C@H]1CC[C@@]1([C@@H]2CC=C2[C@@]1(C)CC[C@@]1([C@H]2CC(C[C@H]1O)(C)C)C)C)C)O[C@@H]1O[C@H](C(=O)O)[C@H]([C@@H]([C@H]1O)O)O	
COc1cc(C=CC(=O)OC2CC[C@]3(C(C2(C)C)CC[C@@]2(C3C=C[C@]34[C@@]2(C)CC[C@@]2(C4C(C)C(C)CC2)C(=O)O3)C)C)ccc1O	(Brezáni et al. 2018)
C[C@@H]1CC[C@@]23[C@@H]([C@H]1C)[C@]1(CC[C@H]4[C@@]([C@@]1(CC2)C)(C)CC[C@@H]1[C@]4(C)CC[C@@H](C1(C)C)O)OC3=O	
C[C@@H]1CC[C@]2([C@@H]([C@H]1C)C1=CC=C3[C@@]([C@@]1(CC2)C)(C)CC[C@@H]1[C@]3(C)CC[C@@H](C1(C)C)O)C(=O)O	
O[C@H]1C[C@@H]2[C@@]3(C)CC[C@@H](C([C@@H]3CC[C@]2([C@]2([C@@]31OC(=O)[C@]1([C@H]3CC(C)(C)CC1)CC2)C)C)(C)C)O	(Poehland et al. 1987)
O=C1CC[C@]2([C@H](C1(C)C)CC[C@@]1([C@@H]2CC[C@H]2[C@@]1(C)CC[C@@H]1[C@]2(C)CC[C@@H]1C(O)(C)C)C)C	
OC[C@H]1O[C@@H](O[C@H]2[C@@H](O[C@@H]([C@H]([C@@H]2O)O)C(=O)O)O[C@H]2CC[C@]3([C@H](C2(C)C)CC[C@@]2([C@@H]3CC=C3[C@@]2(C)CC[C@@]2([C@H]3CC(C)(C)C[C@H]2O)C)C)C)[C@@H]([C@H]([C@@H]1O)O)O[C@@H]1O[C@@H](C)[C@@H]([C@H]([C@H]1O)O)O	(Chapman & Hall Chemical Dictionaries)
OCC1OC(OC2C(OC(C(C2O)O)C(=O)O)OC2CCC3(C(C2(C)C)CCC2(C3CC=C3C2(C)CCC2(C3CC(C)(C)CC2O)C)C)C)C(C(C1O)O)O	
CC[C@H]([C@H]([C@@H]([C@H]([C@H]1CC[C@@H]2[C@]1(C)CC[C@H]1[C@H]2CC(=O)[C@@H]2[C@]1(C)C[C@@H](O)[C@H](C2)O)C)O)O)C(C)C	(Wachsman et al. 2000)

CC[C@H]([C@H]([C@@H]([C@H]([C@H]1CC[C@@H]2[C@]1(C)CC[C@H]1[C@H]2CC(=O)[C@@]2([C@]1(C)C[C@@H](O)[C@H](C2)O)O)C)O)O)C(C)C	
CC(C[C@H]([C@@H]([C@H]([C@H]1CC[C@@H]2[C@]1(C)CC[C@H]1[C@H]2CC(=O)[C@H]2[C@]1(C)CC[C@H](C2)OC(=O)C)C)O)O)C	
CC(C[C@@H]([C@H]([C@H]([C@H]1CC[C@@H]2[C@]1(C)CC[C@H]1[C@H]2CC(=O)[C@@H]2[C@]1(C)CC[C@@H](C2)OC(=O)C)C)O)O)C	
CC(C[C@H]([C@@H]([C@H]([C@H]1CC[C@@H]2[C@]1(C)CC[C@H]1[C@H]2CC(=O)[C@@H]2[C@]1(C)CC[C@@H](C2)Br)C)O)O)C	
CC(C[C@@H]([C@H]([C@H]([C@H]1CC[C@@H]2[C@]1(C)CC[C@H]1[C@H]2CC(=O)[C@@H]2[C@]1(C)CC[C@@H](C2)Br)C)O)O)C	
CC[C@H]([C@@H]([C@H]([C@H]([C@H]1CC[C@@H]2[C@]1(C)CC[C@H]1[C@H]2CC(=O)[C@@]2([C@]1(C)C[C@@H](O)[C@H](C2)O)O)C)O)O)C(C)C	
CC[C@H]([C@@H]([C@H]([C@H]([C@H]1CC[C@@H]2[C@]1(C)CC[C@H]1[C@H]2CC(=O)[C@@]2([C@]1(C)CC[C@@H](C2)Br)O)C)O)O)C(C)C	
CC[C@H]([C@@H]([C@H]([C@H]([C@H]1CC[C@@H]2[C@]1(C)CC[C@H]1[C@H]2CC(=O)[C@@]2([C@]1(C)CC[C@@H](C2)OC(=O)C)O)C)O)O)C(C)C	
CC[C@H]([C@@H]([C@H]([C@H]([C@H]1CC[C@@H]2[C@]1(C)CC[C@H]1[C@H]2CC(=O)[C@@H]2[C@]1(C)C[C@@H](O)[C@H](C2)O)C)O)O)C(C)C	
CC[C@H]([C@H]([C@@H]([C@H]([C@H]1CC[C@@H]2[C@]1(C)CC[C@H]1[C@H]2CC(=O)[C@@]2([C@]1(C)CC[C@@H](C2)Br)O)C)O)O)C(C)C	
CC[C@H]([C@H]([C@@H]([C@H]([C@H]1CC[C@@H]2[C@]1(C)CC[C@H]1[C@H]2CC(=O)[C@@]2([C@]1(C)CC[C@@H](C2)OC(=O)C)O)C)O)O)C(C)C	
C[C@@H]([C@H]1CC[C@@]2([C@]1(C)CC[C@@]13[C@H]2CC[C@@H]2[C@]3(C1)CC[C@@H](C2(C)C)O)C)CC=CC(O)(C)C	(Shamsabadipour et al. 2013)
OC(=O)[C@]12CC[C@@]3([C@@H](C2=CC(CC1)(C)C)CC[C@H]1[C@@]3(C)CC[C@@H]2[C@]1(C)CCC(=O)C2(C)C)C	(Kurokawa et al. 1999)
COC(=O)[C@@]12CC[C@H]([C@@H]2[C@@H]2[C@](CC1)(C)[C@]1(C)CCC3[C@](C1CC2)(C)CC/C(=N\O)/C3(C)C)C(=C)C	(Flekhter et al. 2004)
O/N=C/1\CC[C@]2(C(C1(C)C)CC[C@@]1(C2CC[C@H]2[C@@]1(C)CC[C@]13[C@@H]2[C@@H](OC3=O)C(C)(C)CC1)C)C	

S. 2. The result of cytotoxicity assay at 25 μM.

compound	Cell viability (%)
**1**	116.99 ± 5.20
**2**	116.90 ± 6.99
**3**	111.94 ± 6.85
**4**	108.48 ± 4.17
**5**	121.80 ± 5.60
**6**	122.43 ± 6.84
**7**	114.68 ± 3.95
**8**	122.27 ± 4.78
**9**	105.53 ±3.86
**10**	14.64 ± 2.88**
**10** (50 μM)	1.47 ±0.54**
**11**	111.53 ± 4.45
**11** (50 μM)	113.17 ± 6.08
**12**	111.81 ± 6.79
**12** (50 μM)	111.17 ± 9.73
**13**	104.16 ± 7.71
**14**	112.58 ± 18.62
**15**	114.73 ± 3.93
**16**	129.88 ± 6.83
**16** (50 μM)	2.96 ± 1.77**
**17**	16.84 ±9.50**
**18**	113.47 ± 4.93
**19**	101.34 ± 4.72
ACV (10 μM)	102.92 ± 13.78

S. 3. References

Amoros, M.; Fauconnier, B.; Girre, R. L. (1987): In vitro antiviral activity of a saponin from Anagallis arvensis, Primulaceae, against herpes simplex virus and poliovirus. In *Antiviral Res.* 8 (1), pp. 13–25. DOI: 10.1016/0166-3542(87)90084-2.

Baltina, L. A.; Flekhter, O. B.; Nigmatullina, L. R.; Boreko, E. I.; Pavlova, N. I.; Nikolaeva, S. N. et al. (2003): Lupane triterpenes and derivatives with antiviral activity. In *Bioorganic & Medicinal Chemistry Letters* 13 (20), pp. 3549–3552. DOI: 10.1016/S0960-894X(03)00714-5.

Baltina, L. A., Jr.; Fairushina, A. I.; Baltina, L. A.; Eropkin, M. Yu.; Konovalova, N. I.; Petrova, P. A.; Eropkina, E. M. (2017): Synthesis and Antiviral Activity of Glycyrrhizic-Acid Conjugates with Aromatic Amino Acids. In *Chem. Nat. Compd.* 53 (6), pp. 1096–1100. DOI: 10.1007/s10600-017-2209-7.

Bertol, Jessica Wildgrube; Rigotto, Caroline; Maia de Padua, Rodrigo; Kreis, Wolfgang; Barardi, Celia Regina Monte; Braga, Fernao Castro; Simoes, Claudia Maria Oliveira. (2011): Antiherpes activity of glucoevatromonoside, a cardenolide isolated from a Brazilian cultivar of Digitalis lanata. In *Antiviral Res.* 92 (1), pp. 73–80. DOI: 10.1016/j.antiviral.2011.06.015.

Bloor, S. J.; Qi, L. (1994): Cytotoxic saponins from New Zealand Myrsine species. In *Journal of natural products* 57 (10), pp. 1354–1360. DOI: 10.1021/np50112a004.

Boff, Laurita; Munkert, Jennifer; Ottoni, Flaviano Melo; Zanchett Schneider, Naira Fernanda; Ramos, Gabriela Silva; Kreis, Wolfgang et al. (2019): Potential anti-herpes and cytotoxic action of novel semisynthetic digitoxigenin-derivatives. In *European journal of medicinal chemistry* 167, pp. 546–561. DOI: 10.1016/j.ejmech.2019.01.076.

Brezáni, Viliam; Leláková, Veronika; Hassan, Sherif T. S.; Berchová-Bímová, Kateřina; Nový, Pavel; Klouček, Pavel et al. (2018): Anti-Infectivity against Herpes Simplex Virus and Selected Microbes and Anti-Inflammatory Activities of Compounds Isolated from Eucalyptus globulus Labill. In *Viruses* 10 (7). DOI: 10.3390/v10070360.

Chapman & Hall Chemical Dictionaries: Dictionary of Natural Products. Available online at https://dnp.chemnetbase.com/faces/chemical/ChemicalSearch.xhtml, checked on 1/30/2020.

Cheng, Yuan-Bin; Liao, Tzu-Ching; Lo, Yi-Wen; Chen, Yu-Chen; Kuo, Yuh-Chi; Chen, Shun-Ying et al. (2010): Nortriterpene lactones from the fruits of Schisandra arisanensis. In *Journal of natural products* 73 (7), pp. 1228–1233. DOI: 10.1021/np100048h.

Davola, Maria Eugenia; Mazaira, Gisela I.; Galigniana, Mario D.; Alche, Laura E.; Ramirez, Javier A.; Barquero, Andrea A. (2015): Synthetic pregnenolone derivatives as antiviral agents against acyclovir-resistant isolates of Herpes Simplex Virus Type 1. In *Antiviral Res.* 122, pp. 55–63. DOI: 10.1016/j.antiviral.2015.08.002.

Flekhter, O. B.; Boreko, E. I.; Nigmatullina, L. R.; Pavlova, N. I.; Medvedeva, N. I.; Nikolaeva, S. N. et al. (2004): Synthesis and Pharmacological Activity of Acylated Betulonic Acid Oxides and 28-Oxo-Allobetulone. In *Pharm. Chem. J.* 38 (3), pp. 148–152. DOI: 10.1023/B:PHAC.0000034305.96047.87.

Flekhter, O. B.; Medvedeva, N. I.; Kukovinets, O. S.; Spirikhin, L. V.; Galkin, E. G.; Galin, F. Z. et al. (2007): Synthesis and antiviral activity of lupane triterpenoids with modified cycle E. In *Russ. J. Bioorg. Chem.* 33 (6), pp. 584–588. DOI: 10.1134/S1068162007060088.

Goswami, Debayan; Mahapatra, Ananya Das; Banerjee, Subhadip; Kar, Amit; Ojha, Durbadal; Mukherjee, Pulok K.; Chattopadhyay, Debprasad (2018): Boswellia serrata oleo-gum-resin and β-boswellic acid inhibits HSV-1 infection in vitro through modulation of NF- κ B and p38 MAP kinase signaling. In *Phytomedicine : international journal of phytotherapy and phytopharmacology* 51, pp. 94–103. DOI: 10.1016/j.phymed.2018.10.016.

He, Zhendan; Qiao, Chunfeng; Han, Quanbin; Wang, Ying; Ye, Wencai; Xu, Hongxi. (2005): New triterpenoid saponins from the roots of Platycodon grandiflorum. In *Tetrahedron* 61 (8), pp. 2211–2215. DOI: 10.1016/j.tet.2004.12.032.

Heidary Navid, M.; Laszczyk-Lauer, M. N.; Reichling, J.; Schnitzler, P. (2014): Pentacyclic triterpenes in birch bark extract inhibit early step of herpes simplex virus type 1 replication. In *Phytomedicine : international journal of phytotherapy and phytopharmacology* 21 (11), pp. 1273–1280. DOI: 10.1016/j.phymed.2014.06.007.

Ikeda, Tsuyoshi; Yokomizo, Kazumi; Okawa, Masafumi; Tsuchihashi, Ryota; Kinjo, Junei; Nohara, Toshihiro; Uyeda, Masaru (2005): Anti-herpes virus type 1 activity of oleanane-type triterpenoids. In *Biological & pharmaceutical bulletin* 28 (9), pp. 1779–1781. DOI: 10.1248/bpb.28.1779.

Isaka, Masahiko; Chinthanom, Panida; Srichomthong, Kitlada; Thummarukcharoen, Tuksaporn. (2017): Lanostane triterpenoids from fruiting bodies of the bracket fungus Fomitopsis feei. In *Tetrahedron Lett.* 58 (18), pp. 1758–1761. DOI: 10.1016/j.tetlet.2017.03.066.

Joycharat, Nantiya; Greger, Harald; Hofer, Otmar; Saifah, Ekarin (2008): Flavaglines and triterpenoids from the leaves of Aglaia forbesii. In *Phytochemistry* 69 (1), pp. 206–211. DOI: 10.1016/j.phytochem.2007.06.016.

Khusnutdinova, Elmira F.; Kazakova, Oxana B.; Lobov, Alexander N.; Kukovinets, Olga S.; Suponitsky, Kyrill Yu; Meyers, Craig B.; Prichard, Mark N. (2019): Synthesis of A-ring quinolones, nine-membered oxolactams and spiroindoles by oxidative transformations of 2,3-indolotriterpenoids. In *Organic & biomolecular chemistry* 17 (3), pp. 585–597. DOI: 10.1039/c8ob02624f.

Kinjo, J.; Yokomizo, K.; Hirakawa, T.; Shii, Y.; Nohara, T.; Uyeda, M. (2000): Anti-herpes virus activity of fabaceous triterpenoidal saponins'). In *Biological & pharmaceutical bulletin* 23 (7), pp. 887–889. DOI: 10.1248/bpb.23.887.

Konysheva, A. V.; Tolmacheva, I. A.; Savinova, O. V.; Boreko, E. I.; Grishko, V. V. (2017): Regioselective Transformation of the Cyano Group of Triterpene α,β-Alkenenitriles. In *Chem. Nat. Compd.* 53 (4), pp. 687–690. DOI: 10.1007/s10600-017-2091-3.

Kuo, Ping-Chung; Hwang, Tsong-Long; Lin, Ying-Ting; Kuo, Yuh-Chi; Leu, Yann-Lii. (2011): Chemical constituents from Lobelia chinensis and their anti-virus and anti-inflammatory bioactivities. In *Arch. Pharmacal Res.* 34 (5), pp. 715–722. DOI: 10.1007/s12272-011-0503-7.

Kurokawa, M.; Basnet, P.; Ohsugi, M.; Hozumi, T.; Kadota, S.; Namba, T. et al. (1999): Anti-herpes simplex virus activity of moronic acid purified from Rhus javanica in vitro and in vivo. In *J Pharmacol Exp Ther* 289 (1), pp. 72–78.

Li, Yaolan; Jiang, Renwang; Ooi, Linda S. M.; But, Paul P. H.; Ooi, Vincent E. C. (2007): Antiviral triterpenoids from the medicinal plant Schefflera heptaphylla. In *Phytotherapy research : PTR* 21 (5), pp. 466–470. DOI: 10.1002/ptr.1962.

Liu, Fei; Wang, Ya-Nan; Li, Yong; Ma, Shuang-Gang; Qu, Jing; Liu, Yun-Bao et al. (2017): Rhodoterpenoids A–C, Three New Rearranged Triterpenoids from Rhododendron latoucheae by HPLC–MS–SPE–NMR. In *Scientific reports* 7 (1), p. 7944. DOI: 10.1038/s41598-017-06320-x.

Liu, Fei; Wang, Ya-Nan; Li, Yong; Ma, Shuang-Gang; Qu, Jing; Liu, Yun-Bao et al. (2018): Minor Nortriterpenoids from the Twigs and Leaves of Rhododendron latoucheae. In *Journal of natural products* 81 (8), pp. 1721–1733. DOI: 10.1021/acs.jnatprod.7b01074.

Liu, Fei; Wang, Ya-Nan; Li, Yong; Ma, Shuang-Gang; Qu, Jing; Liu, Yun-Bao et al. (2019): Triterpenoids from the twigs and leaves of Rhododendron latoucheae by HPLC-MS-SPE-NMR. In *Tetrahedron* 75 (2), pp. 296–307. DOI: 10.1016/j.tet.2018.11.059.

Lv, Xiao-Jing; Li, Yong; Ma, Shuang-Gang; Qu, Jing; Liu, Yun-Bao; Li, Yu-Huan et al. (2016): Antiviral Triterpenes from the Twigs and Leaves of Lyonia ovalifolia. In *Journal of natural products* 79 (11), pp. 2824–2837. DOI: 10.1021/acs.jnatprod.6b00585.

Madureira, A. M.; Ascenso, J. R.; Valdeira, L.; Duarte, A.; Frade, J. P.; Freitas, G.; Ferreira, M. J. U. (2003): Evaluation of the antiviral and antimicrobial activities of triterpenes isolated from Euphorbia segetalis. In *Nat. Prod. Res.* 17 (5), pp. 375–380. DOI: 10.1080/14786410310001605841.

Maier, M. S.; Roccatagliata, A. J.; Kuriss, A.; Chludil, H.; Seldes, A. M.; Pujol, C. A.; Damonte, E. B. (2001): Two new cytotoxic and virucidal trisulfated triterpene glycosides from the Antarctic sea cucumber Staurocucumis liouvillei. In *Journal of natural products* 64 (6), pp. 732–736. DOI: 10.1021/np000584i.

Michelini, Flavia M.; Ramírez, Javier A.; Berra, Alejandro; Galagovsky, Lydia R.; Alché, Laura E. (2004): In vitro and in vivo antiherpetic activity of three new synthetic brassinosteroid analogues. In *Steroids* 69 (11-12), pp. 713–720. DOI: 10.1016/j.steroids.2004.04.011.

Mothana, R. A. A.; Awadh Ali, N. A.; Jansen, R.; Wegner, U.; Mentel, R.; Lindequist, U. (2003): Antiviral lanostanoid triterpenes from the fungus Ganoderma pfeifferi. In *Fitoterapia* 74 (1-2), pp. 177–180. DOI: 10.1016/S0367-326X(02)00305-2.

Pei, Ying; Du, Qian; Liao, Peng-Ying; Chen, Zhen-Ping; Wang, Dong; Yang, Chong-Ren et al. (2011): Notoginsenoside ST-4 inhibits virus penetration of herpes simplex virus in vitro. In *Journal of Asian natural products research* 13 (6), pp. 498–504. DOI: 10.1080/10286020.2011.571645.

Petrera, Erina; Joselevich, Maria; Ghini, Alberto; Burton, Gerardo; Coto, Celia E. (2003): Antiherpes virus activities of new 6-19 carbon-bridged steroids and some synthetic precursors. In *Antiviral chemistry & chemotherapy* 14 (5), pp. 243–248. DOI: 10.1177/095632020301400503.

Poehland, B. L.; Carté, B. K.; Francis, T. A.; Hyland, L. J.; Allaudeen, H. S.; Troupe, N. (1987): In vitro antiviral activity of dammar resin triterpenoids. In *Journal of natural products* 50 (4), pp. 706–713. DOI: 10.1021/np50052a022.

Pujol, Carlos A.; Sepúlveda, Claudia S.; Richmond, Victoria; Maier, Marta S.; Damonte, Elsa B. (2016): Polyhydroxylated sulfated steroids derived from 5α-cholestanes as antiviral agents against herpes simplex virus. In *Archives of virology* 161 (7), pp. 1993–1999. DOI: 10.1007/s00705-016-2867-y.

Ryu, Shi Yong; Lee, Chong Kyo; Ahn, Jong Woong; Lee, Seung Ho; Zee, Ok Pyo. (1993): Antiviral activity of triterpenoid derivatives. In *Arch. Pharmacal Res.* 16 (4), pp. 339–342. DOI: 10.1007/BF02977528.

Ryu, Shi Yong; Lee, Chong Kyo; Lee, Chong Ock; Kim, Hae Soo; Zee, Ok Pyo. (1992): Antiviral triterpenes from Prunella vulgaris. In *Arch. Pharmacal Res.* 15 (3), pp. 242–245. DOI: 10.1007/BF02974063.

Shamsabadipour, Sara; Ghanadian, Mustafa; Saeedi, Hojjatollah; Rahimnejad, Mohammad Reza; Mohammadi-Kamalabadi, Marzieh; Ayatollahi, Syed Majid; Salimzadeh, Loghman. (2013): Triterpenes and steroids from Euphorbia denticulata Lam. with anti-herpes symplex virus activity. In *Iran. J. Pharm. Res.* 12 (4), pp. 759–767.

Simões, C. M.; Amoros, M.; Girre, L. (1999): Mechanism of antiviral activity of triterpenoid saponins. In *Phytotherapy research : PTR* 13 (4), pp. 323–328. DOI: 10.1002/(SICI)1099-1573(199906)13:4<323::AID-PTR448>3.0.CO;2-C.

Sotanaphun, Uthai; Lipipun, Vimolmas; Yaipakdee, Panadda; Bavovada, Rapepol. (2005): New acidic-rearranged compounds from tinge none derivatives and their biological activity. In *Pharm. Biol. (Philadelphia, PA, U. S.)* 43 (1), pp. 39–46. DOI: 10.1080/13880200590903345.

Su, Chun-Ting; Hsu, John T.-A.; Hsieh, Hsing-Pang; Lin, Pi-Han; Chen, Ting-Chi; Kao, Chuan-Liang et al. (2008): Anti-HSV activity of digitoxin and its possible mechanisms. In *Antiviral Res.* 79 (1), pp. 62–70. DOI: 10.1016/j.antiviral.2008.01.156.

Tolmacheva, I. A.; Igosheva, E. V.; Savinova, O. V.; Boreko, E. I.; Grishko, V. V. (2014): Synthesis and antiviral activity of C-3(C-28)-substituted 2,3-seco-triterpenoids. In *Chem. Nat. Compd.* 49 (6), pp. 1050–1058. DOI: 10.1007/s10600-014-0821-3.

Tolmacheva, I. A.; Igosheva, E. V.; Vikharev, Yu. B.; Grishko, V. V.; Savinova, O. V.; Boreko, E. I.; Eremin, V. F. (2013): Synthesis and biological activity of mono- and diamides of 2,3-secotriterpene acids. In *Russ. J. Bioorg. Chem.* 39 (2), pp. 186–193. DOI: 10.1134/S1068162013020143.

Tolmacheva, Irina A.; Igosheva, Ekaterina V.; Savinova, Olga V.; Boreko, Eugene I.; Eremin, Vladimir F.; Grishko, Victoria V. (2019): Synthesis and evaluation of antiviral activities of triterpenic conjugates with 2-aminobutan-1-ol as potent microbicidal agents. In *Med. Chem. Res.* 28 (10), pp. 1648–1660. DOI: 10.1007/s00044-019-02401-w.

Tolmacheva, Irina A.; Nazarov, Alexey V.; Dmitriev, Maxim V.; Boreko, Eugene I.; Grishko, Victoria V. (2017): Synthesis of 1,10-seco-triterpenoids by the Beckmann fragmentation from allobetulin. In *Tetrahedron* 73 (45), pp. 6448–6455. DOI: 10.1016/j.tet.2017.09.044.

Visalli, Robert J.; Ziobrowski, Hannah; Badri, Kameswara R.; He, Johnny J.; Zhang, Xiugen; Arumugam, Sri Ranjini; Zhao, Hua (2015): Ionic derivatives of betulinic acid exhibit antiviral activity against herpes simplex virus type-2 (HSV-2), but not HIV-1 reverse transcriptase. In *Bioorganic & Medicinal Chemistry Letters* 25 (16), pp. 3168–3171. DOI: 10.1016/j.bmcl.2015.05.099.

Wachsman, Monica B.; Lopez, Elsa M. F.; Ramirez, Javier A.; Galagovsky, Lydia R.; Coto, Celia E. (2000): Antiviral effect of brassinosteroids against herpes virus and arenaviruses. In *Antiviral chemistry & chemotherapy* 11 (1), pp. 71–77. DOI: 10.1177/095632020001100107.

Wachsman, Monica B.; Ramirez, Javier A.; Talarico, Laura B.; Galagovsky, Lydia R.; Coto, Celia E. (2004): Antiviral activity of natural and synthetic brassinosteroids. In *Current Medicinal Chemistry - Anti-Infective Agents* 3 (2), pp. 163–179. DOI: 10.2174/1568012043354026.

Wang, Weihong; Li, Famei; Alam, Naseer; Liu, Yonghong; Hong, Jongki; Lee, Chong-Kyo et al. (2002): New saponins from the starfish Certonardoa semiregularis. In *Journal of natural products* 65 (11), pp. 1649–1656. DOI: 10.1021/np020234r.

Yoneda, Taichi; Nakamura, Seikou; Ogawa, Keiko; Matsumoto, Tomoko; Nakashima, Souichi; Matsumura, Kiriko et al. (2018): Oleanane-type Triterpenes with Highly-Substituted Oxygen Functional Groups from the Flower Buds of Camellia sinensis and Their Inhibitory Effects against NO Production and HSV-1. In *Nat. Prod. Commun.* 13 (2), 1934578X1801300206. DOI: 10.1177/1934578x1801300206.

Zhao, Xiao-Tong; Yu, Mei-Hua; Su, Shi-Yun; Shi, Xun-Long; Lei, Chun; Hou, Ai-Jun (2020): Cycloartane triterpenoids from Pseudolarix amabilis and their antiviral activity. In *Phytochemistry* 171, p. 112229. DOI: 10.1016/j.phytochem.2019.112229.

Zígolo, M. Antonela; Salinas, Maximiliano; Alché, Laura; Baldessari, Alicia; Liñares, Guadalupe García (2018): Chemoenzymatic synthesis of new derivatives of glycyrrhetinic acid with antiviral activity. Molecular docking study. In *Bioorganic chemistry* 78, pp. 210–219. DOI: 10.1016/j.bioorg.2018.03.018.
